# NMDA receptors are selectively partitioned into complexes and supercomplexes during synapse maturation

**DOI:** 10.1038/ncomms11264

**Published:** 2016-04-27

**Authors:** René A. W. Frank, Noboru H. Komiyama, Tomás J. Ryan, Fei Zhu, Thomas J. O'Dell, Seth G. N. Grant

**Affiliations:** 1 Centre for Clinical Brain Sciences, University of Edinburgh, Chancellor's Building, 49 Little France Crescent, Edinburgh EH16 4SB, UK; 2 MRC Laboratory of Molecular Biology, Hills Road, Cambridge CB2 0QH, UK; 3 RIKEN-MIT Center for Neural Circuit Genetics at the Picower Institute for Learning and Memory, Department of Biology, Massachusetts Institute of Technology, Cambridge, Massachusetts 02139, USA; 4 Department of Brain and Cognitive Sciences, Massachusetts Institute of Technology, Cambridge, Massachusetts 02139, USA; 5 Howard Hughes Medical Institute, Massachusetts Institute of Technology, Cambridge, Massachusetts 02139, USA; 6 Department of Physiology, David Geffen School of Medicine and UCLA Integrative Center for Learning and Memory, University of California Los Angeles, Los Angeles, California 90095, USA

## Abstract

How neuronal proteomes self-organize is poorly understood because of their inherent molecular and cellular complexity. Here, focusing on mammalian synapses we use blue-native PAGE and ‘gene-tagging' of GluN1 to report the first biochemical purification of endogenous NMDA receptors (NMDARs) directly from adult mouse brain. We show that NMDARs partition between two discrete populations of receptor complexes and ∼1.5 MDa supercomplexes. We tested the assembly mechanism with six mouse mutants, which indicates a tripartite requirement of GluN2B, PSD93 and PSD95 gate the incorporation of receptors into ∼1.5 MDa supercomplexes, independent of either canonical PDZ-ligands or GluN2A. Supporting the essential role of GluN2B, quantitative gene-tagging revealed a fourfold molar excess of GluN2B over GluN2A in adult forebrain. NMDAR supercomplexes are assembled late in postnatal development and triggered by synapse maturation involving epigenetic and activity-dependent mechanisms. Finally, screening the quaternary organization of 60 native proteins identified numerous discrete supercomplexes that populate the mammalian synapse.

## Abstract (web summary)

NMDARs and MAGUK proteins are capable of forming higher-order protein assemblies, however their organisation in the intact brain is unclear. Here, Frank *et al*. identify mouse and human supercomplexes and discover their mechanism of assembly using genetic tagging and affinity purification.

## Graphical abstract

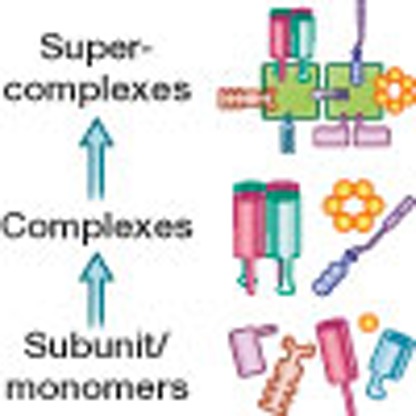

Synapses contain highly complex proteomes that when disrupted by mutations cause over 100 brain diseases^1^,^2^. Since the function of almost all cellular proteins is dependent on their higher-order assembly into ‘molecular machines'^3^,^4^, it is imperative to characterize the self-assembly of synapse proteins and the impact of mutations. However, defining the organization of the myriad synapse proteins is poorly understood largely because of the technical challenges imposed by the cellular and molecular complexity of the brain.

Canonical models of the higher-order assembly of proteins are hierarchical (Fig. 1a) with individual constituent subunits forming oligomeric complexes that may further associate into mega-Dalton sized supercomplexes (complexes of complexes)^5^. Amongst the most highly studied examples of synaptic multiprotein machines are those comprising the *N*-methyl-D-aspartic acid receptors (NMDAR) and Dlg/MAGUK proteins, which are key mediators of synaptic plasticity, cognitive functions and implicated in multiple brain disorders^6^,^7^,^8^,^9^,^10^,^11^. The finding that PDZ-ligands in GluN2 subunits of NMDARs bind the PDZ domains of Dlg/MAGUK proteins has underpinned the general paradigm that neurotransmitter receptors and other classes of synaptic proteins require this interaction for the purpose of anchoring and assembly with additional proteins^12^,^13^,^14^,^15^. However, it is not known if the promiscuity of PDZ interactions is sufficient for assembly *in vivo* or if other mechanisms select for the assembly of specific combinations of synaptic proteins^16^.

The study of higher-order protein assemblies in mammals is further complicated by the evolution of more complex synapse proteomes in the vertebrate lineage^17^. A major feature distinguishing vertebrate from invertebrate synapses is the expansion in proteome complexity that arose following two genome duplications in early chordates^18^,^19^,^20^. The increase in number of subunits may have had a multiplicative impact on the diversity of potential vertebrate complexes and supercomplexes. This is exemplified by the NMDAR-Dlg/MAGUK assembly: many invertebrate genomes encode a single-GluN2 and -Dlg protein^21^, whereas mammals express four, each retaining the conserved PDZ binding capacity and thereby permitting 16 combinations of GluN2-Dlg/MAGUK interactions. In the absence of existing methods that enable the efficient purification of intact native NMDAR-Dlg/MAGUK complexes from the mammalian brain, the actual diversity of vertebrate complexes/supercomplexes remains unknown. Furthermore, whether the redundant PDZ-ligand interactions confer resilience to mutations in individual GluN2 or Dlg paralogs is unknown because of the lack of studies testing paralog mutations on complex formation. These issues are fundamental to our understanding of the molecular organization of the synapse as well as the interpretation of the growing body of literature that has identified disease-causing mutations in NMDAR-Dlg/MAGUK complexes and other postsynaptic proteins^2^,^22^.

To address these issues we developed a biochemical and mouse genetic approach to characterize the supermolecular organization of proteomes *in vivo*, focusing on native NMDAR complexes. First, using a simple biochemical screen we identify that all native NMDAR from the mouse forebrain are partitioned between ∼0.8 and ∼1.5 MDa complexes. Next, we report the knock-in of a high-affinity peptide tag into the gene encoding GluN1 subunit of the NMDAR in mouse. This enables the purification of all endogenous NMDARs, as well as the identification and quantification of constituents. Subunit tagging combined with multiple other mutant mice revealed a novel tripartite mechanism for assembly between NMDAR, PSD95 and PSD93 supercomplexes. This mechanism regulates assembly late in postnatal development. Supercomplex assembly is also driven by synapse maturation involving epigenetic and activity-dependent palmitoylation. Finally, we used a biochemical screen to survey 60 diverse proteins from within the synapse proteome identifying their higher-order organization into 190 novel complexes and supercomplexes consistent with those containing NMDARs. These findings reveal specific mechanisms that restrict the association of synapse proteins into specific supercomplexes and identify a role in synapse maturation.

## Results

### Two supermolecular NMDAR complexes in mice and humans

**Figure 1:**
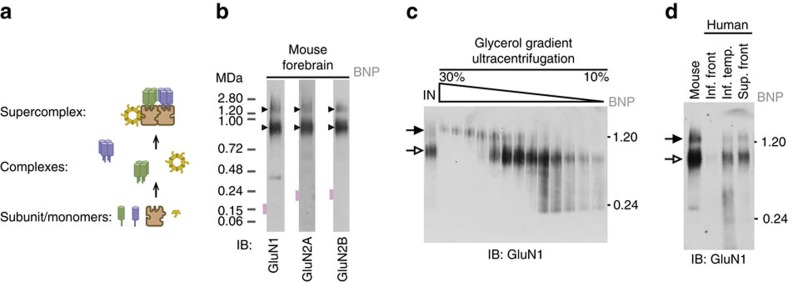
Native supermolecular assembly of NMDAR subunits in the mammalian brain. (**a**) Schematic showing the higher-order assembly from individual proteins to complexes and supercomplexes (complexes of complexes). (**b**) Native protein complexes of GluN1, GluN2A and GluN2B detected in BNP immunoblot screen of mouse forebrain extracts. Approximately 0.8 and ∼1.5 MDa complexes indicated by filled and open arrowheads, respectively (hereafter used to label all figures). The expected size of each protein in monomeric form indicated with pink rectangle. On left side, non-denaturing molecular mass indicated in mega-Daltons (MDa). (**c**) BNP GluN1 immunoblot of fractions from glycerol gradient (10–30%) ultracentrifugation. ‘IN', forebrain extract supernatant. On right side, non-denaturing molecular mass indicated in MDa. (**d**) BNP GluN1 immunoblot of fresh human cortical biopsy samples from the inferior frontal (inf. front.), inferior temporal (inf. temp.) and superior frontal (sup. front.) lobes. Mouse forebrain extract supernatant shown for comparison. These data show the ∼0.8 and ∼1.5 MDa NMDA receptor complexes (1.5-NR and 0.8-NR) were conserved between mouse and humans.

Numerous reports using co-immunoprecipitation and *in vitro* methods have shown that NMDARs are associated with upwards of 50 different synaptic proteins, which are expected to affect the Ca^2+^-dependent synaptic signalling, anchoring and trafficking of NMDARs. How these apparently numerous binary interactions relate to the assembly of native NMDARs is unknown. For example, does the native forebrain NMDAR complex contain all of these components all of the time? To address this question we employed blue-native polyacrylamide gel electrophoresis (BNP)^23^ to explore the size distribution of the total population of NMDARs in the mouse forebrain. In contrast to co-immunoprecipitation, this permitted us to identify the distinct complexes within which each brain protein reside, without requiring prior knowledge of their constituents. BNPs showed native NMDARs (containing GluN1, GluN2A and GluN2B) were distributed between only two distinct complexes with apparent median masses of ∼840 kDa and ∼1.5 MDa (Fig. 1b), for simplicity hereafter referred to as 0.8-NR and 1.5-NR, respectively. Although the detergent-lipid micelle surrounding 0.8-NR and 1.5-NR likely contributes to the observed masses, both were also separable on the basis of size by ultracentrifugation through a glycerol gradient (Fig. 1c), confirming these molecular species were distinct. The existence of NMDARs in these two forms has not been reported previously and we therefore examined whether both were detectable in human brain. BNP immunoblots probed with GluN1 (Fig. 1d) confirmed both 0.8-NR and 1.5-NR in extracts from neurosurgical biopsies of the human neocortex. These data suggest that two distinct supermolecular forms differing in size constitute native NMDARs of the adult mammalian forebrain.

### Isolation of native NMDAR complexes from *Glun1* ^*TAP*^ mice

**Figure 2:**
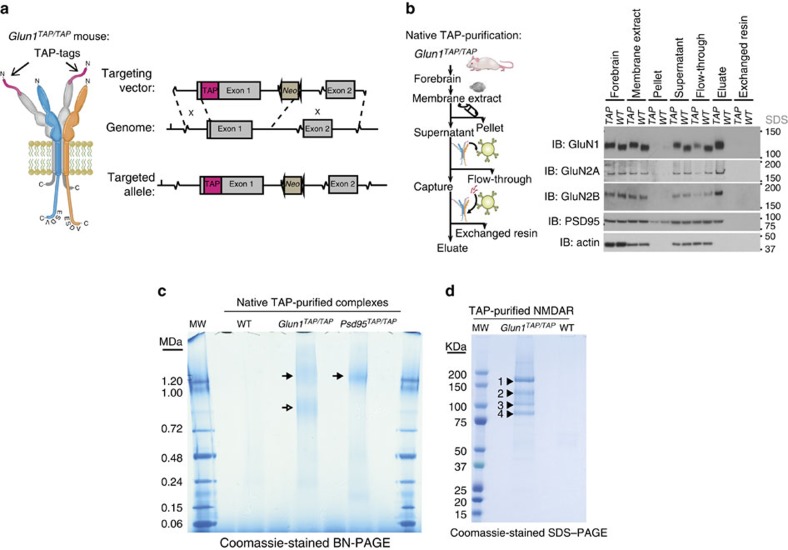
**Generation of TAP-tagged GluN1 (*****Glun1***^***TAP/TAP***^**) knock-in mice for purification of native NMDARs.** (**a**) Genetic engineering of TAP-tags into GluN1. Left, schematic shows tetrameric NMDAR with two GluN1 subunits (grey) engineered with tandem affinity peptide (TAP-tags, magenta) on their extracellular N-termini. GluN2 subunits (GluN2A, cyan; GluN2B, orange) shown with C-terminal cytoplasmic ESDV motifs/PDZ-ligands. TAP-tag encodes 3xFlag and His_x6_. Right, schematic shows gene-targeting vector carrying TAP-tag sequence in *Glun1* exon1, 5′ and 3′ regions of homology with genome and resultant targeted allele below. The neomycin selection cassette was subsequently deleted using Cre/loxP. Grey-filled boxes, exons; magenta, TAP cassette; brown box, *neo* neomycin resistance cassette; brown triangle, *loxP* site; dotted lines, homology arms. (**b**) Purification of native NMDARs from *Glun1*^*TAP/TAP*^ mouse forebrains. Left, schematic of purification steps and samples corresponding to right, immunoblots of NMDAR subunits, PSD95, actin. Dissected mouse forebrains were homogenized and fractionated. Crude membrane fraction was solubilized (membrane extract) and separated by centrifugation (supernatant and pellet). Supernatant was incubated with Flag-affinity resin capturing TAP-tagged receptors with some residual Flow-through. High yields of native receptor were released (eluate) by peptide-antigen exchange. Following elution no material remained (exchanged resin). Right, purification from *Glun1*^*TAP/TAP*^ (TAP) and WT control mouse shows receptor subunits (GluN1, GluN2A, GluN2B), PSD95, and actin detected by SDS–PAGE immunoblots from indicated fractions. Note the higher molecular weight of TAP-engineered GluN1 compared with WT. IB, immunoblotting antibody; MW, molecular weight markers. SDS, SDS–PAGE. (**c**) Coomassie-stained BNP of TAP-purified NMDAR and PSD95 complexes from *Glun1*^*TAP/TAP*^ and *Psd95*^*TAP/TAP*^ mice, respectively. TAP-purified complexes isolated from forebrain extracts from control (WT), *Glun1*^*TAP/TAP*^ and *Psd95*^*TAP/TAP*^ mice were separated on BNP gel and Coomassie stained. Supplementary Fig. 2a shows excised bands used in native proteomic analysis (TAP-BNP-MS). Filled arrow indicates ∼1.5 MDa complexes and open arrow indicates 0.8-NR. Molecular weight in MDa shown on left and in stained ladder in first and last gel lanes. (**d**) Coomassie-stained SDS–PAGE of TAP-purified NMDARs from *Glun1*^*TAP/TAP*^ and control (WT) mice. Abundant constituents of bands 1–4 were identified by MALDI-MS: GluN2A/B, TAP-GluN1, PSD93 and PSD95, respectively. MW shown in kDa on left.

To understand the molecular basis for the partitioning of NMDARs into two supermolecular forms requires identification of constituents that distinguish 0.8-NR from 1.5-NR. Purification of scarce native assemblies from complex tissues typically suffers from low yields and the absence of reliable controls. To avert this problem, we used mouse genetics to insert a tandem affinity purification (TAP) tag into the first protein-coding exon of the GluN1 subunit gene (*Glun1*) by homologous recombination in embryonic stem cells (Fig. 2a). Because most proteomic methods rely on denaturation to elute purified complexes, which prevents individual complexes from being separated from one another, we included a 3xFlag tag. This design permitted gentle elution (by competition with 3xFlag peptide) of intact individual complexes for further separation and characterization (Supplementary Fig. 1a).

Following germline transmission of this TAP-GluN1 knock-in mutation (*Glun1*^*TAP/+*^), mice were intercrossed to produce viable *Glun1*^*TAP/TAP*^ homozygotes. Next, these mice were examined in biochemical, anatomical and electrophysiological assays, which indicated that the TAP-tagged NMDAR functioned normally (Supplementary Fig. 1b–g). Optimization of the purification conditions resulted in almost complete solubilization of TAP-GluN1, GluN2A and GluN2B, thus providing material that was representative of essentially all endogenous NMDARs (Fig. 2b, lanes 5 and 6 from left and Supplementary Fig. 2a). A single step of Flag-affinity purification (Fig. 2b and Supplementary Fig. 2b) yielded 10–25 μg protein from five adult mice, which corresponds to ∼10^12^ receptors per mouse forebrain.

Purified TAP-NMDARs complexes were directly visible as coomassie-stained BNP bands equivalent to those detected in forebrain extract by BNP immunoblot (Fig. 2c, left lane), indicating the native NMDARs were purified intact. No bands were visible in control (wild type (WT)) samples (Fig. 2c, left lane), indicating the specificity of the purification. To determine the molecular constituents that might differentiate 0.8-NR from 1.5-NR, we excised each BNP band and profiled their composition using mass spectrometry (BNP-MS, Supplementary Table 1). A sample of these MS-identified proteins was validated by BNP immunoblot (Supplementary Fig. 2c,d), suggesting the 0.8-NR band was composed solely of NMDAR subunits and represents the expected ‘classical' tetrameric channel complex. In contrast, the 1.5-NR band contained the NMDAR complex in association with 50 other proteins (see Supplementary Table 1 for full list) including other ion channel complexes (Kir2.3), trans-synaptic adhesion (Adam22), scaffold (PSD95, PSD93) and signalling proteins (CaMKIIα). Thus, the size and composition suggest 1.5-NR represent a sub-population of NMDAR supercomplexes.

To explore the higher-order assembly of synaptic receptor complexes further we used the same TAP-tag and BNP-MS strategy to analyse an available *Psd95*^*TAP/TAP*^ knock-in mice line^24^ and found TAP-purified PSD95 was readily visible on coomassie-stained BNP within a single ∼1.5 MDa band (Fig. 2c, right lane). Thus, essentially all PSD95 assembles into ∼1.5 MDa complexes (hereon called 1.5-PSD95). Moreover, MS of the gel-excised 1.5-PSD95 band identified 89 proteins, of which 87% were the same as those in 1.5-NR (Supplementary Table 2). The constituents of 1.5-NR and 1.5-PSD95 were consistent with the numerous proteins reported to interact with either NMDAR or PSD95 (refs 24, 25). Importantly, these associations shown here were all confined within a discrete subset of NMDARs in the mega-Dalton size range, which is inconsistent with models of these synaptic proteins co-assembled into either a single very large complex or distributed within a network.

We therefore reasoned that the majority of 1.5-NR constituents would be in low abundance. Accordingly, Coomassie-staining of purified NMDAR separated by SDS-polyacrylamide gel electrophoresis (PAGE) revealed only four major bands (with apparent molecular weights of 180, 130, 105 and 85 kDa), and many more bands of lower abundance (Fig. 2d). The principal constituents of the four abundant bands, identified by peptide mass fingerprinting, were GluN2A/GluN2B, TAP-GluN1, PSD93 and PSD95, respectively. These data support a model in which there are many different 1.5-NR complexes each with different constituents and also suggest a central role for PSD95 and PSD93. We confirmed the minimal requirements for the assembly of 0.8-NR and 1.5-NR by heterologous expression of subunits in HEK cells. Transient transfection of HEK293 cells with complementary DNAs encoding GluN1 and GluN2B in HEK293 produced 0.8-NR (Supplementary Fig. 2e), whereas transient transfection with complementary DNAs encoding GluN1, GluN2B, PSD95 and PSD93, produced 0.8-NR and 1.5-NR (Supplementary Fig. 2f). Other constituents of forebrain 1.5-NR are likely to increase the range of masses as shown by BNP immunoblot of proteins co-purified with TAP-GluN1 (Supplementary Fig. 2d). To test the specificity and mechanism of assembly we next used mouse genetics to mutate constituents of 1.5-NR *in vivo*.

### A tripartite mechanism for the assembly of ∼1.5 MDa NMDARs

**Figure 3:**
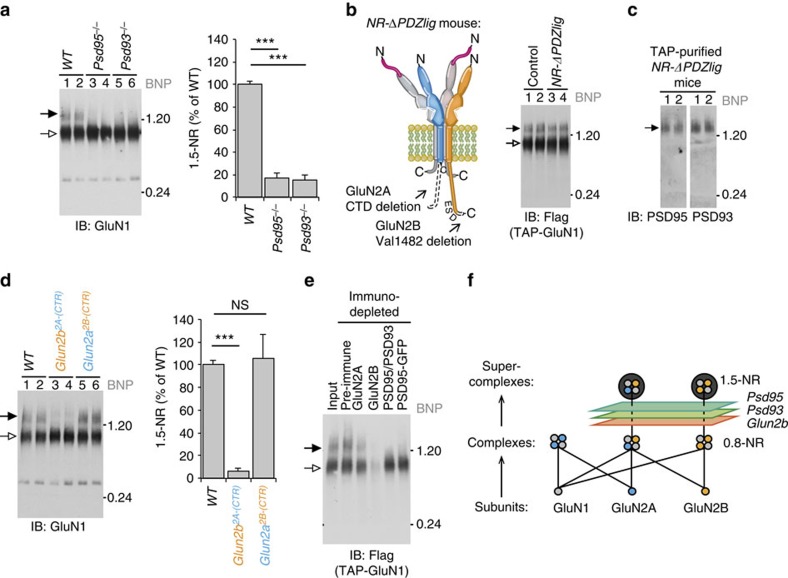
Mutant mouse screen of NMDAR supermolecular assembly. (**a**) Left, BNP GluN1 immunoblot of forebrain extracts WT (lanes 1 and 2, duplicates), *Psd95*^*−/−*^ (lanes 3 and 4), *Psd93*^*−/−*^ (lanes 5 and 6). Right panel, quantification shows relative to WT (*n*=6) 1.5-NR decreased to 17% (*P*<0.001) and 15% (*P*<0.001) in *Psd95*^*−/−*^ (*n*=6) and *Psd93*^*−/−*^ (*n*=6) mice, respectively. (**b**) Assembly of 1.5-NR does not require conserved PDZ-ligands. Left, schematic of NMDAR in *NR-ΔPDZlig* mice (*Glun2b*^*dV/dV*^*/Glun2a*^*dC/dC*^*/Glun1*^*TAP/TAP*^). The receptor lacks the GluN2A CTD and terminal valine of GluN2B ESDV motif and contains the TAP-tagged GluN1. GluN1 subunit, grey; TAP-tags, magenta; GluN2A subunit, cyan; GluN2B subunit, orange. Right, BNP immunoblot of *NR-ΔPDZlig* forebrain extracts (lanes 3 and 4) and control (*Glun1*^*TAP/TAP*^; lanes 1 and 2) mice with Flag antibody detecting TAP-GluN1. Sample load was normalized by total NMDAR concentration (Supplementary Fig. 4b). (**c**) PSD95 and PSD93 assemble with NMDARs in 1.5-NR from *NR-ΔPDZlig* mice. 1.5-NR was TAP-purified *NR-ΔPDZlig* mice (shown as duplicate lanes labelled 1 and 2) and BNPs immunoblotted with antibodies to PSD95 (left panel) and PSD93 (right panel). (**d**) Assembly of 1.5-NR requires GluN2B CTD and does not require GluN2A CTD. Left, BNP GluN1 immunoblot of forebrain extracts from WT (lanes 1 and 2, duplicates), *Glun2b*^*2A(CTR)/2A(CTR)*^ (lanes 3 and 4), *Glun2a*^*2B(CTR)/2B(CTR)*^ (lanes 5 and 6) mice. Cyan and orange labels indicate chimeric *Glun2a* and *Glun2b* knock-in mutations, respectively^20^. Right, quantification shows in *Glun2b*^*2A(CTR)/2A(CTR)*^ mice 1.5-NR decreased to 6% (*t*-test, *P*<0.001) of WT (*n*=6). (**e**) Immuno-depletion of GluN2B or PSD95 removes all 1.5-NR. Extracts from *Glun1*^*TAP/TAP*^*/Psd95*^*EGFP/EGFP*^ double knock-in mice were subunit-depleted with antibodies (shown in lanes) then separated on BNP for immunoblotting with Flag antibody to detect NMDARs. Lanes; input, total extract; immuno-depleting antibodies (from left to right): non-specific IgG, GluN2A, GluN2B and GFP. See Supplementary Fig. 4c for controls. (**f**) Schematic summary of tripartite genetic requirements of *Glun2b, Psd95 and Psd92* for the assembly of ∼1.5 MDa NMDAR supercomplexes. GluN2A and GluN2B subunits assemble into three 0.8-NR subtypes (complexes of GluN2A di-heteromers, GluN2A/GluN2B tri-heteromers, GluN2B di-tetramers). GluN1 subunit, grey; GluN2A subunit, cyan; GluN2B subunit, orange. BNP, blue-native PAGE. Molecular weight in MDa shown on right. Error bars indicate s.e.m. Representative results from triplicate experiments shown. IB, immunoblot.

On the assumption that *in vitro* GluN2 binds interchangeably with either PSD95 or PSD93 via the same PDZ-ligand^12^,^13^ we reasoned that a knockout of either MAGUK would not prevent NMDAR recruitment into ∼1.5 MDa supercomplexes. However, Fig. 3a shows that in *Psd95*^*−/−*^ knockout mice 1.5-NR was essentially absent from the whole forebrain. Perhaps more intriguing was the discovery that in *Psd93*^*−/−*^ knockout mice 1.5-NR also failed to assemble (Fig. 3a). Thus, the interaction of NMDAR with either PSD95 or PSD93 was not sufficient to recruit NMDARs into ∼1.5 MDa complexes; instead *both* PSD95 and PSD93 were essential.

These genetic interdependencies for 1.5-NR assembly were highly selective because in *Psd95*^*−/−*^ mice 1.5-PSD93 remain and in *Psd93*^*−/−*^ most 1.5-PSD95 were still present (Supplementary Fig. 3). This result also indicates that a large population of PSD95 and PSD93 must exist in complexes that are independent of NMDARs.

One potential explanation for the dual genetic dependency of PSD95 and PSD93 for 1.5-NR assembly is that the PDZ-ligand interactions with GluN2A/B are not sufficient *in vivo*. To test directly the necessity of PDZ-ligands *in vivo* we engineered double mutant mice that lacked both GluN2A and GluN2B PDZ-ligands in their C-termini. First, we targeted a point mutation that deletes the C-terminal valine from the gene encoding GluN2B, producing *Glun2b*^*dV*^ mice (Supplementary Fig. 4a). *Glun2b*^*dV/dV*^ homozygous mice were viable and fertile. Next, to render forebrain NMDARs completely devoid of C-terminal PDZ-ligands on both GluN2A and GluN2B, we crossed *Glun2b*^*dV/dV*^ with another available line in which the entire carboxy-terminal domain (CTD) of GluN2A was deleted (*Glun2a*^*dC/dC*^ mice)^26^. The resulting *NR*-Δ*PDZlig* double knock-in mice were crossed with *Glun1*^*TAP*^ to produce *Glun2b*^*dV/dV*^*/Glun2a*^*dC/dC*^*/Glun1*^*TAP/TAP*^ triple homozygous knock-in mutants that enable purification of receptors from *NR*-Δ*PDZlig* mice (Fig. 3b, left). Remarkably, in *NR*-Δ*PDZlig* mice the assembly of NMDARs into 1.5-NR was preserved (Fig. 3b, right). We confirmed the association between NMDARs, PSD95 and PSD93 in the absence of PDZ-ligands by detecting PSD95 and PSD93 in 1.5-NR purified from *NR*-Δ*PDZlig* mice (Fig. 3c). Although the PDZ-ligand was not essential for the assembly of 1.5-NR, we observed a notable reduction in the total expression levels of all NMDAR subunits (GluN1, GluN2A and GluN2B) in *NR*-Δ*PDZlig* mice (Supplementary Fig. 4b), suggesting the PDZ-ligand influences receptor biogenesis or trafficking^27^.

Having established that the PDZ-ligand interaction was dispensable, we reasoned that the mechanism of NMDAR assembly with MAGUKs could be mediated by sequences other than the conserved ESDV motif within the GluN2 CTDs (ref. 28). The CTD is the most divergent domain between GluN2A and GluN2B paralogs^21^, raising the possibility that only one of these two paralogs was capable of directing assembly of NMDARs into 1.5-NR. To test the role of GluN2A and GluN2B CTDs we examined two knock-in mutant mice lines. In the first mutant, *Glun2b*^*2A(CTR)/2A(CTR)*^, mice express only GluN2A CTDs (the exon encoding the GluN2B CTD has been replaced with a copy of the exon that encodes the GluN2A CTD)^20^.

In the forebrains of homozygous *Glun2b*^*2A(CTR)/2A(CTR)*^ mice the NMDAR was almost completely absent from the ∼1.5 MDa supercomplexes, leaving only 0.8-NR (Fig. 3d). Thus, GluN2A CTDs were insufficient to replace GluN2B CTDs for assembling NMDARs with PSD95 in 1.5-NRs. In accordance, in *Glun2a*^*2B(CTR)/2B(CTR)*^ knock-in mice that only express the GluN2B CTD (the exon encoding the GluN2B CTD is inserted into the GluN2A gene) we found NMDARs assembled into both ∼1.5 and ∼0.8 MDa complexes (Fig. 3d and Supplementary Fig. 3). Thus, the CTD of GluN2B specifically assembles NMDAR-Dlg/MAGUK supercomplexes independent of its PDZ-ligand.

These genetic findings make the prediction that the higher-order assembly of receptor tetramers into 1.5-NRs is not promiscuous because it is essential for any given tetramer to contain a minimum of one GluN2B subunit; those receptors containing only GluN2A subunits will be excluded from the population of 1.5-NRs (Fig. 3d). We tested this biochemically by depleting all GluN2B-containing receptors from extracts and, as predicted, BNP immunoblots showed no detectable 1.5-NR (Fig. 3e, immunodepletion controls in Supplementary Fig. 4c). Indeed, most receptors including 0.8-NR were also removed, indicating the majority of native NMDARs contained at least one GluN2B in the adult forebrain. In contrast, subunit-depleting GluN2A-containing complexes, removed only a small fraction of 1.5-NR (Fig. 3e), which corresponded to heterotetramers containing a single GluN2A and GluN2B subunit.

Similarly, when both PSD95 and PSD93 were immuno-depleted, all detectable 1.5-NR was removed from the sample (Fig. 3e), while the amount of 0.8-NR remained unaffected. To deplete just PSD95 and rule out the possibility that PSD95 antibodies were non-specific, we used a knock-in mouse carrying an in-frame, C-terminal GFP-tag targeted to the gene encoding PSD95 (*Psd95*^*EGFP/EGFP*^, reported elsewhere). Immuno-depleting GFP from the brain extracts of *Psd95*^*EGFP/EGFP*^*-Glun1*^*TAP/TAP*^ double knock-in mice also removed all 1.5-NR (Fig. 3e). Therefore, every 1.5-NR must contain at least one molecule of PSD95. Together these genetic and biochemical results indicate a mechanism of 1.5-NR assembly that was applicable across the mouse forebrain and required three components: PSD95, PSD93 and the CTD of GluN2B (Fig. 3f).

### GluN2B is fourfold more abundant than GluN2A in forebrain

**Figure 4:**
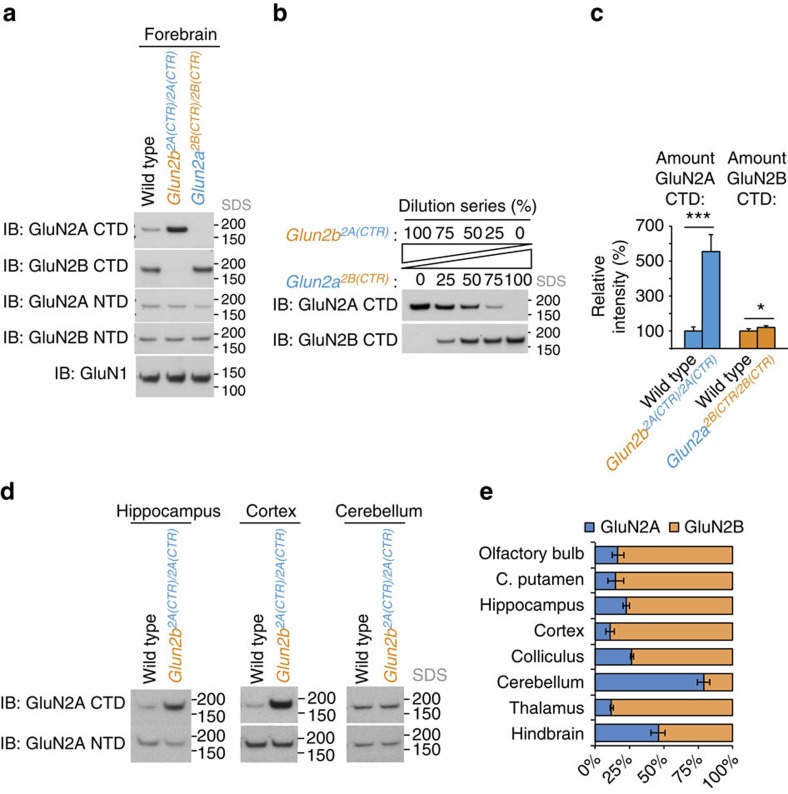
The GluN2A/GluN2B relative molar abundance using mouse genetics and quantitative tagging. (**a**) SDS–PAGE immunoblot of WT, *Glun2b*^*2A(CTR)/2A(CTR)*^ and *Glun2a*^*2B(CTR)/2B(CTR)*^ forebrain extract. Top panel, detected the GluN2A C-terminal domain (CTD). Second panel down, GluN2B CTD. *Glun2a*^*2B(CTR)/2B(CTR)*^ and *Glun2b*^*2A(CTR)/2A(CTR)*^ provided negative controls for the specificities of the GluN2A CTD and GluN2B CTD antibodies, respectively. Third, fourth and fifth panels down, detected GluN2A NTD, GluN2B NTD subunits and GluN1, respectively. Blue and orange labels indicate chimeric *Glun2a* and *Glun2b* knock-in mutations, respectively^20^. (**b**) Dilution series of *Glun2a*^*2B(CTR)/2B(CTR)*^ forebrain extract into that of *Glun2b*^*2A(CTR)/2A(CTR)*^ indicated sensitivity of quantification. Immunoblots detected GluN2A CTD (upper panel) and GluN2B CTD (lower panel). (**c**) Quantification of GluN2A CTD and GluN2B CTD immunoblots. Measurements from *Glun2b*^*2A(CTR)/2A(CTR)*^ (*P*<0.001) and *Glun2a*^*2B(CTR)/2B(CTR)*^ (*t*-test*, P*<0.02) were normalized to WT. (**d**) SDS–PAGE immunoblot of WT, *Glun2b*^*2A(CTR)/2A(CTR)*^ extracts from left to right: cortex, hippocampus and cerebellum. Top panels, detected the GluN2A C-terminal domain (CTD). Lower panels, detected GluN2A NTD. (**e**) Quantification of GluN2A relative to GluN2B in eight different brain regions from top to bottom: olfactory bulb, caudate putamen, hippocampus, cortex, colliculus, cerebellum, thalamus and hindbrain. GluN2A was measured using GluN2A CTD antibodies against WT extracts (*n*=3). GluN2B was measured by measuring GluN2A CTD antibodies against *Glun2b*^*2A(CTR)/2A(CTR)*^ extracts (*n*=3) and subtracting the intensity from that of WT. Error bars indicate 1 s.d.

The immuno-depletion experiments indicated that almost all NMDARs contain a GluN2B subunit and that very few contain GluN2A (Fig. 3e), yet prevailing models suggest GluN2A is the most abundant GluN2 subunit in the adult forebrain^7^,^29^,^30^ or that the major population of NMDARs are triheteromeric^11^,^31^,^32^. To investigate this discrepancy further, a genetic tagging assay was devised to measure the molar ratio of GluN2A/GluN2B expression *in vivo* using the *Glun2b*^*2A(CTR)/2A(CTR)*^ knock-in mice. First, we showed by immunoblotting the N-terminal domain that replacing the CTD of GluN2B with that of GluN2A in *Glun2b*^*2A(CTR)/2A(CTR)*^ mice caused no change in the total expression levels of GluN2A, GluN2B or GluN1 compared with WT (Fig. 4a, bottom three panels). Next, we measured in total forebrain extracts the relative amount of the GluN2A CTD and found it was fivefold greater in *Glun2b*^*2A(CTR)/2A(CTR)*^ compared with WT (Fig. 4a, top panel). This indicates that the molar ratio of GluN2A to GluN2B was 1:4±1 (mean±s.d.) in the adult forebrain (Fig. 4b,c and Methods section ‘Quantification of subunit molar ratios *in vivo'*).

As a further control, we performed the reciprocal measurement of the GluN2B CTD in *Glun2a*^*2B(CTR)/2B(CTR)*^ mice^20^. In accordance with the observation that GluN2A subunits were expressed at much lower abundance than GluN2B in the adult forebrain, the amount of GluN2B detected with antibodies that recognize the GluN2B CTD was only slightly greater in *Glun2a*^*2B(CTR)/2B(CTR)*^ compared with WT (Fig. 4a, second from top panel, Fig. 4b–c).

Different brain regions might have different molar ratios of GluN2A to GluN2B that could skew the overall average; therefore, we measured the molar ratio of GluN2A to GluN2B within eight distinct brain regions of the adult mouse. As expected, GluN2A was more abundant than GluN2B within the cerebellum (Fig. 4d,e). In the cortex and hippocampus, GluN2B was six- and threefold more abundant than GluN2A, respectively. In the hindbrain, GluN2A and GluN2B were present at near equimolar amounts. In all other brain regions a three- to sixfold excess of GluN2B over GluN2A was detected (Fig. 4e). Quantification of the excess of GluN2B over GluN2A is therefore widespread and consistent with the importance of GluN2B for the assembly of 1.5 MDa supercomplexes in the forebrain.

### Supercomplex assembly in synapse maturation

Having established the composition and genetic dependencies, we next sought to understand the functional context of NMDAR and PSD95 supercomplexes. An extensive literature has highlighted the importance of NMDAR and PSD95 in the postnatal development and synapse maturation of the mammalian brain^33^. We hypothesized that the two NMDAR complexes found in the adult brain may be subject to differential regulation during development.

**Figure 5:**
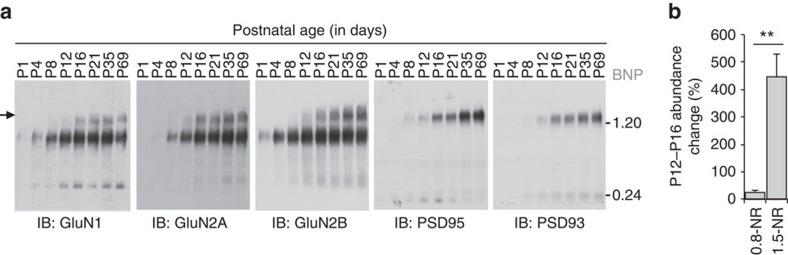
1.5-NR and 1.5-PSD95 assemble late in postnatal development. (**a**) BNP immunoblots show WT mouse forebrain extracts at developmental time points probed with antibodies against GluN1, GluN2A, GluN2B, PSD95 and PSD93. Filled arrow, ∼1.5 MDa complexes that only assembly from ∼P16 onwards. Sample load was normalized by forebrain mass. Molecular weight in MDa shown on right. (**b**) Quantification of changing abundance of 0.8-NR and 1.5-NR from P12 (*n*=3) to P16 (*n*=3) using BNP immunoblots of GluN1. 0.8-NR increased by 25%, whereas 1.5-NR increased by 445%; *t*-test, *P*>0.01; Error bars indicate s.e.m.

We compared the native assembly of NMDAR complexes at multiple postnatal ages, from birth (P1) to adulthood (P69) in the mouse forebrain, a period marked by profound synaptogenesis and synapse maturation. At all ages we observed 0.8-NR complexes (Fig. 5a), whereas significant amounts of 1.5-NR were seen only from P16 onwards (Fig. 5b). In keeping with the tripartite mechanism of assembly, 1.5-PSD95 and 1.5-PSD93 were absent in the first postnatal week and increase significantly from the second postnatal week onwards (Fig. 5a). Thus, 0.8-NR is constitutively expressed at all ages, whereas 1.5-NR assembly is developmentally regulated and characteristic of the mature forebrain.

**Figure 6:**
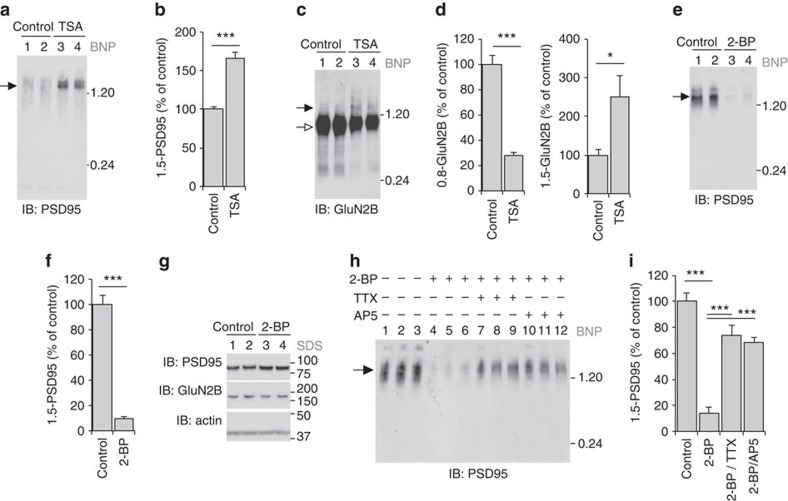
1.5-NR and 1.5-PSD95 assembly is correlated with synapse maturation. (**a**) HDAC1/2 inhibition with TSA causes increased assembly if 1.5-PSD95. Cultured primary cortical neurons (DIV7) were treated with DMSO (control; lanes 1 and 2) 0.25 μM TSA (lanes 3 and 4) or for 18–24 h before cells were harvested and analysed by BNP PSD95 immunoblot. (**b**) Quantification shows TSA treatment increased 1.5-PSD95 to 166% (*n*=6) of control. *t*-test, *P*<0.001; Error bars indicate s.e.m. (**c**) HDAC1/2 inhibition causes decreased and increased assembly of 0.8-NR and 1.5-NR, respectively. Cultured primary cortical neurons (DIV7) were treated with DMSO (control; lanes 1 and 2) 0.25 μM TSA (lanes 3 and 4) or for 18–24 h before cells were extracted and analysed by BNP GluN2B immunoblot. (**d**) Left, quantification shows TSA treatment decreased 0.8-NR to 28% (*n*=6) of control. *P*<0.001; Error bars indicate s.e.m. Right, TSA treatment increased 1.5-NR to 251% (*n*=6) of controls. *P*<0.05; error bars indicate s.e.m. (**e**) Inhibition of palmitoylation with 2-bromopalmidate (2-BP) decreased 1.5-PSD95. Cultured primary cortical neurons (DIV14) were treated with DMSO (control; lanes 1 and 2) 10 μM 2-BP (lanes 3 and 4) for 8 h before cells were extracted and analysed by BNP PSD95 immunoblot. (**f**) Quantification shows 2-BP treatment decreased 1.5-PSD95 to 10% (*n*=6) of control. *P*<0.001; error bars indicated s.e.m. (**g**) Inhibition of palmitoylation with 2-BP does not change the expression levels of PSD95 or GluN2B. (**h**) 2BP-mediated disassembly of 1.5-PSD95 is activity dependent. Cultured primary cortical neurons (DIV14) were treated with DMSO (control; lanes 1, 2 and 3) 10 μM 2-BP (lanes 4, 5 and 6), 10 μM 2-BP with 1 μM TTX (lanes 7, 8 and 9), 10 μM 2-BP with 50 μM APV (lanes 10, 11 and 12) for 8 h before cells were extracted and analysed by BNP PSD95 immunoblot. Filled arrow indicates 1.5-PSD95. Molecular weight in MDa shown on right. (**i**) Quantification shows 2-BP treatment decreased 1.5-PSD95 to 14% of control (*n*=6, *P*<0.001). TTX rescued 2-BP-dependent disassembly (*n*=6, *P*<0.001) to 74% of control. AP5 rescued 2-BP-dependent disassembly (*n*=6, *P*<0.001) to 69% of control; error bars indicated s.e.m.

We therefore wondered if the late postnatal re-organization of synaptic proteins into supercomplexes was a property of synapse maturation^34^,^35^,^36^,^37^,^38^. Epigenetic mechanisms involving histone deacetylases have been reported to regulate synapses maturation^39^, stabilize memory and enhance memory retrieval in mouse models of Alzheimer's disease^40^,^41^. To test the role of supermolecular assembly in synapse maturation we used an inhibitor of HDAC1/2, trichostatin A (TSA), in primary neuronal cultures^39^,^42^. We found DIV7 primary neuronal cells, similar to the early postnatal forebrain, contain only trace amounts of 1.5-NR. Following 18–24 h TSA treatment, we observed a significant recruitment of PSD95 from its monomeric form into 1.5-PSD95 supercomplexes over control cells (Fig. 6a,b). This TSA-stimulated synapse maturation also caused a significant decrease of 0.8-NR and increase of 1.5-NR (Fig. 6c,d). These findings suggest epigenetic mechanisms can regulate the assembly of 1.5-NR and 1.5-PSD95 during synapse maturation.

The punctate distribution of PSD95 that is characteristic of mature synapses can be reversed using inhibitors of palmitoylation^43^. To test a structural role of palmitoylation in 1.5-PSD95 assembly we chemically de-palmitoylated proteins in forebrain extracts by nucleophilic thiolysis (with 40 mM MESNA or 40 mM β-mercaptoethanol)^44^, which had no effect on the assembly 1.5-PSD95 (Supplementary Fig. 5a). Thus, palmitoylation *per se* is not necessary for maintaining PSD95 within 1.5 MDa supercomplexes. Since palmitoylation also regulates trafficking^45^, we tested if instead palmitoylation had an indirect influence on the assembly of 1.5-PSD95. Treating DIV14 primary neurons for 8 h with an inhibitor of palmitoylation, 2-bromopalmitate (2BP) caused a dramatic disassembly of 1.5-PSD95 (Fig. 6g,f), without affecting total expression of PSD95 (Fig. 6g). The effect of 2BP on 1.5-NR could not be determined because primary neurons cultured from 14 to 28 days contain only trace amounts of 1.5-NR compared with neurons within the intact forebrain (Fig. 6c). The 2BP-dependent decrease in 1.5-PSD95 was rescued by blocking activity with 1 μM TTX or inhibiting NMDARs with 50 μM AP5 (Fig. 6h,i), suggesting activity-dependent palmitoylation cycling^43^ regulates the dynamics of 1.5-PSD95 assembly. The 2BP-dependent decrease in 1.5-PSD95 coincided with a reduction in the punctate distribution of PSD95 (Supplementary Fig. 5b) as previously reported^43^. Together these results show the late postnatal assembly of supercomplexes is determined by the tripartite mechanism and can be modulated by epigenetic, palmitoylation and activity-dependent processes.

### Higher-order molecular architecture of the synapse

**Figure 7:**
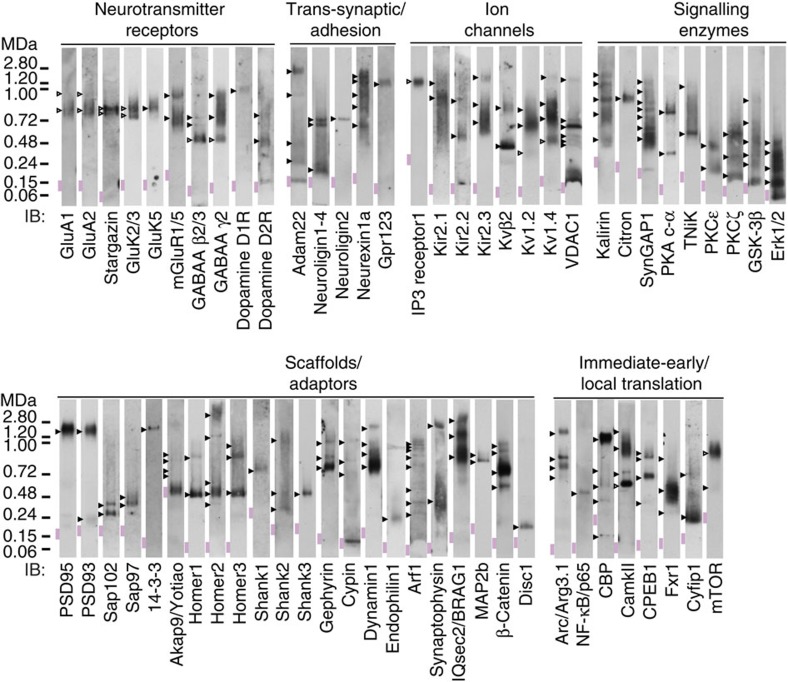
Screen of native protein assemblies of 60 brain proteins detected by BNP (blue non-denaturing PAGE) immunoblot of mouse forebrain extracts. Complexes were extracted with a panel of five different buffers. For most candidate proteins, different buffers extracted complexes of a similar size (see Methods section for details and Supplementary Data 1 for list of all complexes). Novel and previously described complexes indicated by filled and open arrowheads, respectively. The expected size of each protein in monomeric form indicated with pink rectangle. Non-denaturing molecular mass indicated in mega-Daltons (MDa).

Finally, we asked if the organizational principles identified for NMDARs were consistent with the higher-order assembly of 60 diverse proteins within the synaptic proteome. Total mouse forebrain synaptoneurosomes were solubilized in a screen of five buffers (with different detergents of varying stringency), then rapidly resolved by blue non-denaturing-PAGE (BNP), and proteins detected by immunoblotting (see Methods section for details). As shown in Fig. 7, we examined proteins from six major functional modalities of the synapse: neurotransmitter receptors, trans-synaptic/adhesion, ion channels, signalling enzymes, scaffolds/adaptors and immediate-early/local translation.

Strikingly, every protein was tethered in at least one complex, typically 5–20x its monomeric size, consistent with expectations that almost all individual proteins function within higher-order assemblies^4^. After correcting for the potential over-estimation of mass (≤100 kDa) as a consequence of the contribution of the detergent/lipid micelle, we found only 13 bands corresponded to the size of native proteins unassembled in monomeric form. This left 220 reproducible and separable protein bands that likely represent the major populations of the candidate proteins. Each band was assigned as a complex and compared with previously published results (Supplementary Data 1). Seventeen bands corresponded to the size of known oligomeric complexes including multiple native AMPA receptor and auxiliary subunits that co-migrated in ∼700 kDa complexes^46^. The remaining 190 bands were many times larger than the monomeric size of the protein, indicating the existence of many novel complexes or supercomplexes. As expected from the MS analysis of TAP-purified NMDAR and PSD95, we observed 15 constituent proteins including Kir2.3, Arc and Iqsec2 with bands co-migrating at ∼1.5 MDa molecular weight (Fig. 7 and listed in Supplementary Data 1, column D, orange), indicating a large fraction of the population these proteins assemble into supercomplexes.

Lastly, we examined the complexes from a subset of proteins within our screen (mGluR1/5, Arc, β-catenin and NF-κB) at multiple postnatal ages between P1 and P69 (Supplementary Fig. 5c) revealing several different temporal profiles of supermolecular assembly, and thus suggesting the regulated assembly of synaptic supercomplexes is a widespread property of postnatal development.

## Discussion

This integrative biochemical and genetic analysis identifies mechanisms controlling the higher-order assembly of proteins within the mammalian synapse proteome and novel supermolecular forms of the NMDAR. Endogenous NMDARs were partitioned within two discrete populations of 0.8 and 1.5 MDa, which were biochemically, genetically and developmentally separable. The former was expressed at all postnatal ages and the latter first appeared late in postnatal development and driven by synapse maturation. Genetic dissection of the two supermolecular forms using a battery of mutant mice revealed 1.5-NR assembly was critically dependent on a tripartite requirement of PSD95, PSD93 and GluN2B. A screen of the quaternary organization of 60 additional synapse-associated proteins revealed 190 separable complexes and supercomplexes within the mouse forebrain that were congruent with the constituents 1.5-NR.

Surprisingly, three findings were inconsistent with the prevailing model that 1.5-NR assembly is mediated by C-terminal PDZ-ligands binding to Dlg/MAGUK proteins. First, we found the GluN2A and GluN2B ESDV ligands were not required *in vivo*. Second, we found that both PSD95 and PSD93 were necessary (either MAGUK alone was not sufficient for the assembly of NMDAR with PSD95). Third, we found that the cytoplasmic domain of GluN2A could not assemble with PSD95 and/or PSD93, whereas GluN2B alone was sufficient (independent of its PDZ-ligand). Although our findings do not support the PDZ-ligand-dependent assembly of NMDARs with Dlg/MAGUK proteins, the reduced overall levels of NMDAR expression in mice lacking GluN2A/B PDZ-ligands indicates the importance of this interaction in the biogenesis or trafficking of the receptor before assembly with Dlg/MAGUKs into synaptic supercomplexes. Moreover, both the developmental and genetic investigations of 0.8-NR and 1.5-NR show that the PDZ-ligand interaction with Dlg/MAGUKs is not required for synaptic localization of NMDARs: 1.5-NRs were absent during the first 12 postnatal days (when NMDA function is essential) and the three mutants lacking 1.5-NRs in adulthood have all been reported to have normal synaptic NMDAR currents in hippocampus CA1 region^20^,^47^,^48^,^49^.

Functional insight was apparent from the regulated assembly of 1.5-NR, and 1.5-PSD95 late in postnatal development from ∼P12 onwards. This time point coincides with eye opening and a period of marked synaptogenesis within the forebrain^33^,^50^,^51^. Consistent with this developmental regulation we also identified a role for 1.5-NR and 1.5-PSD95 in synapse maturation. Blockade of histone deacetylases in immature primary neurons that has been reported to accelerate synapse maturation^39^, increased the assembly of 1.5-NR and 1.5-PSD95. These data indicate that epigenetic mechanisms influence the assembly of synaptic supercomplexes.

A defining property of maturation is the punctate enrichment of PSD95 within the postsynaptic membrane, which is regulated by palmitoylation cycles^43^. Blockade of palmitoylation signalled an activity-dependent disassembly of 1.5-PSD95, which as previously reported^43^, caused a dramatic decrease in PSD95 puncta. Thus the present data suggest 1.5-PSD95 supercomplexes are the molecular species responsible for the punctate appearance of PSD95 that indicates a functionally mature synapse^52^.

The quantification of GluN2A and GluN2B within populations of postsynaptic complexes and supercomplexes impact on models that suggest differential functions for these subunits^7^,^11^. We have shown that *in vivo* the adult forebrain contains fourfold molar excess of GluN2B over GluN2A. Most GluN2A was assembled with GluN2B (in so called triheteromeric receptors)^31^,^53^ and GluN2A is dependent on GluN2B for assembly into ∼1.5 MDa NMDAR-MAGUK supercomplexes. Moreover, we expect further subpopulations of these complexes since additional receptors, ion channels, signalling and trans-synaptic adhesion proteins were detected as constituents of 1.5-NRs and in discrete 1.5 MDa complexes by BNP immunoblot (Fig. 7). The brain region-specific differences in the molar ratio of GluN2A to GluN2B raise the possibility that supercomplexes with different compositions are allocated into anatomically distinct synapses.

This study provides new insight into the higher-order molecular organization of the synapse proteome. Almost all surveyed proteins were found in novel complexes and supercomplexes. It was also striking that many related proteins (within the same paralog family) did not all co-migrate, suggesting they were organized in separable complexes (Fig. 7). For example, the four members of the MAGUK/Dlg family have each been reported to interact promiscuously with numerous synaptic proteins (including NMDAR subunits). However, in our screen we found MAGUKs were differentially organized with PSD95 and PSD93 in genetically separable 1.5 MDa supercomplexes, whereas the largest population of Sap97/Dlg1 and Sap102/Dlg3 were retained within smaller (0.2–0.4 MDa) complexes. Similar patterns emerge in comparing proteins within 10 out of 12 paralog families surveyed in our screen (Fig. 7 and Supplementary Data 1). Interestingly, paralog-mediated restriction together with the modular arrangement of proteins apparent within our BNP screen (Fig. 7) are two mechanisms that diminish the combinatorial molecular complexity of the synapse and thereby overcome a theoretical ‘complexity brake' on systems with high numbers of components such as the brain^3^,^54^,^55^.

Although many disease-associated mutations have been reported in proteins co-purifying in the 1.5 MDa TAP-NR and TAP-PSD95 supercomplexes (including GluN2A, GluN2B and PSD93/Dlg2)^2^,^22^, this study provides the first direct evidence that mutations disrupt their supermolecular organization at the synapse. We found the 1.5-NRs were exquisitely sensitive to mutations in PSD95, PSD93 and GluN2B. There is abundant evidence of physiological and behavioural changes in mice carrying mutations in these genes^19^,^20^,^47^,^48^,^56^,^57^. This is particularly compelling for mutations in PSD95 since we showed most PSD95 resides within 1.5 MDa assemblies. In humans, PSD93 mutations also cause cognitive impairments in touchscreen tests of cognition, impacting on the same forms of learning, cognitive flexibility and attention as those seen in PSD93 mutant mice^19^. Recurrent *de novo* mutations in PSD93 and GluN2 genes are now widely reported in schizophrenia as are mutations in the sets of proteins isolated in 1.5-NR and 1.5-PSD95 complexes^19^,^58^,^59^,^60^. Thus, future studies that dissect the postsynaptic complexes could shed light on the synaptic mechanisms of brain disorders.

## Methods

### BNP screen of synaptic complexes

Forebrains (hippocampus and cortex) were dissected from adult (P56–70) mice and were homogenized (12 strokes with a Teflon-glass pestle and mortar) in 5 ml ice-cold buffer H (1 mM Na HEPES pH7.4, 320 mM sucrose with protease inhibitors). The homogenate pellet was collected by centrifugation with 1,000*g*. (MLA-80, 5,000 r.p.m.) at 2 °C for 10 min and re-homogenized (6 strokes) in 2 ml buffer H and centrifuged as before. The first and second 1,000*g*. supernatants were pooled and centrifuged at 18,500*g*. (MLA80, 19,000 r.p.m.) to pellet the crude membranes. The 18,500*g*. supernatant was discarded. Extraction conditions were screened using the crude membrane from 50 to 60 mg mouse forebrain re-suspended in 0.5 ml buffer H, to which different detergents in 0.5 ml buffer X (100 mM NaCl, 50 mM tris.Cl pH8) were mixed for 1 h at 6–10 °C. Final detergent concentrations were 1% w/v. Detergents used: sodium deoxycholate (Figs 1, 2, 3 and 5, 6, 7), 3-[(3-cholamidopropyl)dimethylammonio]-1- propanesulfonate (Fig. 6), n-Dodecyl-*N*,*N*-Dimethyl-3-Ammonio-1-Propanesulfonate (Fig. 7), β-D-maltopyranoside (Fig. 7), (Fig. 7). Next, insoluble proteins were removed from the total extract by centrifugation at 120,000 *g*. (MLA-80, 50,000 r.p.m.) for 40 min at 8 °C. BNPs were immediately run and immunoblotted to detect the native complexes of NMDAR subunits (Fig. 1b) and 60 additional brain proteins (Fig. 7). In general different detergent buffers gave similarly sized complexes and the less stringent detergent buffers were less efficient at solubilizing native complexes. Antibodies and all the native complexes detected with each detergent buffer are detailed in Supplementary Data 1.

### BNP immunoblot and immuno-depletions

BNP was run according to Schägger^23^. For immuno-depletions, extract supernatant were incubated with antibodies to deplete subunits at 6–10 °C for 2–16 h. To capture antibody-antigen complexes, 3 mg protein G magnetic beads were added for 30 min at 6–10 °C. Additional immunoblots ensured the completeness of immuno-depletions (Supplementary Fig. 4c).

### Generation of *Glun1*^*TAP/TAP*^ knock-in mice

Sequence encoding a TAP-tag was designed for in-frame insertion within exon 1 of the Glun1 gene, downstream of the predicted signal peptidase cleavage site (Asp22) and before the predicted start of the amino-terminal domain (Pro23). The tag contained a linker (-PSGSTG-), a 3xFlag epitope tag (-DYKDHDIDYKDDDDK-), linker containing a TEV protease consensus site (-GTENVLYFQGT-), hexa-histidine tag (-GRSHHHHHH-) and a linker to the beginning of the amino-terminal domain (-GAS-). The 161-bp sequence encoding the TAP tag with flanking restriction sites (*Age*I and *Nhe*I) was synthesized by ligase chain reaction. Upstream of the tag, 2,249 bp 5′ homology arm was retrieved by recombineering with 500 bp mini-homology arms and mouse genomic BAC clone (bMQ-32D16) in *E. coli* EL350. The mini-homology arms flanked by restriction sites (*Not*I and *Sca*I) were synthesized by PCR from mouse genomic BAC clone. Downstream of the TAP-tag, the 3′ flank comprising the 3′ end of exon 1 (280 bp) and the first 500 bp of intron 1 were retrieved with flanking restriction sites (*Nhe*I and *Sbf*I) by PCR from mouse genomic BAC clone. Additional restriction sites (*Nde*I and *Bsr*GI) suitable for genomic southern genotyping were also included upstream of the *Sbf*I restriction site in this product. The 5′ homology arm, TAP-tag and 3′ flank products were assembled sequentially within an intermediate vector by restriction (*Not*I/*Sca*I, *Sca*I/*Nhe*I and *Nhe*I/*Sbf*I) and ligation. This product assembly was placed upstream of a 2-kbp neomycin selection cassette flanked by lox P sites and restriction sites (*Sbf*I and *Spe*I) within the intermediate vector. The product assembly together with the lox P-flanked *Neomycin* cassette (2,200 bp) was cut (*Not*I, *Spe*I) from the intermediate vector and ligated into the targeting vector, which also contained a 5-kb 3′ homology arm. This homology arm was built from 500 bp mini homology arms flanked by restriction sites (*Spe*I and *Hin*dIII), which were synthesized by nested PCR from a mouse genomic BAC clone and ligated into the targeting vector. The 3′ mini homology arms were used to retrieve the 3′ homology arms from the mouse genomic BAC clone by recombineering in *E. coli* EL350. The targeting vector also contained a Diphtheria Toxin A cassette for negative selection of non-homologous integration.

The linearized targeting vector was electroporated into E14Tg2a ES cells. ES cell clones mutated by homologous recombination were identified from genomic DNA samples by PCR. Six out of 216 ES clones were putative recombinants. Recombination was confirmed for 2 of these 6 clones by genomic southern hybridization to regions outside the 5′ and 3′ homology arms of *Nde*I digested genomic DNA. Positive ES clones were microinjected into C57BL/6 blastocysts, which generated chimeras containing 30–70% mutant cells. Chimeras back-crossed onto 129S5 gave germ line transmission, which was genotyped by PCR and sequenced from ear biopsy samples.

The neomycin selection cassette that was inserted into the first intron of *Glun1* (Fig. 2a, right) was removed by backcrossing on a transgenic *Cre* recombinase-expressing mouse. Double mutant mice (homozygous at both alleles) containing *Glun1*^*TAP/TAP*^ with other mutations were also bred. Except for developmental experiments, all mice were adult, age-, gender- and background-matched.

### Purification of native NMDARs from TAP-tagged knock-in mice

The crude membranes from 5 P56-P70 *Glun1*^*TAP/TAP*^ mouse forebrains were re-suspended in 12.5 ml buffer H and extracted with 12.5 ml 2% deoxycholate, 100 mM NaCl, 50 mM Tris.Cl pH8 for 1 h at 6 °C. Total extract was centrifuged at 120,000*g*. for 40 min at 8 °C. Conditions for immuno-capture, wash and peptide-antigen exchange elution were screened using a high-throughput purification robot (MAGic sample processor, Invitrogen). For 25 ml *Glun1*^*TAP/TAP*^ extract supernatant, 80 μg mouse Flag antibody was coupled to 30 mg (500 μl) protein G magnetic beads (Invitrogen). Receptor was captured from extract supernatant for 2 h. The beads were washed three times with 5 ml wash buffer (0.37% w/v sodium deoxycholate, 0.05 mg.ml^−1^ lipids (1:1:3 POPC:POPE:POG), 150 mM NaCl, 50 mM Tris.Cl pH8). Flag captured complexes were eluted with 600 μl wash buffer supplemented with 0.2 mg.ml^−1^ Flag peptide for 2 h at 6 °C. Eluate was buffer exchanged to remove the Flag elution peptide and concentrated with a 100-kDa MWCO centrifugal filter (Amicon) to 35 μl 0.1–0.25 mg.ml^−1^ native NMDAR.

### Native proteomics (TAP-BNP-LC-MS/MS)

Excised BNP gel slices containing the purified proteins were prepared for mass spectrometric analysis by manual *in situ* enzymatic digestion. Briefly, the excised protein gel pieces were placed in a well of a 96-well microtitre plate and de-stained with 50% v/v acetonitrile and 50 mM ammonium bicarbonate, reduced with 10 mM DTT and alkylated with 55 mM iodoacetamide. After alkylation, proteins were digested with 6 ng μl^−1^ Trypsin (Promega, UK) overnight at 37 °C. The resulting peptides were extracted in 2% v/v formic acid, 2% v/v acetonitrile. The digest was analysed by nano-scale capillary LC-MS/MS using an Ultimate U3000 HPLC (ThermoScientific Dionex, San Jose, USA) to deliver a flow of ∼300 nl min^−1^. A C18 Acclaim PepMap100 5 μm, 100 μm × 20 mm nanoViper (ThermoScientific Dionex, San Jose, USA), trapped the peptides before separation on a C18 Acclaim PepMap100 3 μm, 75 μm × 150 mm nanoViper (ThermoScientific Dionex, San Jose, USA). Peptides were eluted with a gradient of acetonitrile. The analytical column outlet was directly interfaced via a modified nano-flow electrospray ionization source, with a hybrid dual pressure linear ion trap mass spectrometer (Orbitrap Velos, ThermoScientific, San Jose, USA). Data-dependent analysis was carried out, using a resolution of 30,000 for the full MS spectrum, followed by 10 MS/MS spectra in the linear ion trap. MS spectra were collected over an *m*/*z* range of 300–2,000. MS/MS scans were collected using a threshold energy of 35 for collision induced dissociation. LC-MS/MS data were then searched against a protein database (UniProt KB) using the Mascot search engine programme (Matrix Science, UK)^61^. Database search parameters were set with a precursor tolerance of 5 p.p.m. and a fragment ion mass tolerance of 0.8 Da. Two missed enzyme cleavages were allowed and variable modifications for oxidized methionine, carbamidomethyl cysteine, pyroglutamic acid, phosphorylated serine, threonine, tyrosine, tert-butyloxycarbonyl-lysine, norbornene-lysine and prop-2-yn-1-yloxycarbonyl-lysine were included. MS/MS data were validated using the Scaffold programme (Proteome Software Inc., USA). All data were additionally interrogated manually. Peptide identifications were accepted if they could be established at >80.0% probability as specified by the Peptide Prophet algorithm^62^. Protein probabilities were assigned by the Protein Prophet algorithm^62^. Using these stringent identification parameters, the rate of false-positive identifications is <1%. All non-mouse proteins that were detected but could not be matched to a mouse protein (in Swissprot-TrEMBL) were not included in the analysis below (foe example, human keratins).

The composition of ∼1.5 MDa complexes from TAP-GluN1, PSD95-TAP and WT control were compared (Supplementary Table 1) using a stringent threshold of detection (⩾2 unique peptides and ⩾1 unique peptide in ⩾2 replicate samples). This level of stringency was sufficient to detect 55 proteins in TAP-GluN1, 89 proteins in PSD95-TAP and only one protein in the WT control (IgG). Since it expected that the ‘tails' of the bands may partially overlap, the differential composition of proteins in the ∼0.8 and ∼1.5 MDa TAP-GluN1 bands were validated by BNP immunoblot of TAP-purified complexes (Supplementary Fig. 2d).

All MS data (raw data, mascot files and scaffold file) were processed with the PRIDE converter^63^ and deposited in the PRIDE database (http://www.ebi.ac.uk/pride/)^64^ with ProteomeXchange accession number: PXD000011.

### MALDI-MS

To identify the principle NMDAR constituents (Fig. 2d), 35 μl concentrated TAP-purified samples were denatured in SDS loading buffer and separated on a 4–12% bis-tris polyacrylamide gel. The gel was fixed and stained with colloidal Brilliant Blue-G Coomassie (Sigma). The principle bands were identified by MALDI-MS mass fingerprinting. Gel bands were excised and subjected to the following treatment (30 min per step, 20 °C, in 100 mM ammonium bicarbonate/50% acetonitrile): (1) Reduction with 5 mM tris(2-carboxyethyl)phosphine. (2) Alkylation by addition of iodoacetamide (25 mM final concentration) and (3) removal of liquid then wash. Gel pieces were dried *in vacuo* for 10 min and 25 μl 100 mM ammonium bicarbonate containing 10 μg ml^−1^ modified trypsin (Promega) was added. Digestion was for 17 h at 32 °C. Peptides were recovered and desalted using uC18 ZipTip (MIllipore) and eluted to a MALDI target plate using 2 μl α-cyano-4-hydroxycinnamic acid matrix (Sigma) in 50% acetonitrile/0.1% trifluoroacetic acid. Peptide mass were determined using a MALDI micro MX mass spectrometer (Waters) in reflection mode and analysed with Masslynx software. Database searches of the mass fingerprint data were performed using Mascot (http://www.matrixscience.com).

### Generation of *Glun2b* ^*2BdV*^ knock-in mice

The *Glun2b*^*2BdV*^ knock-in mouse was engineered by standard mouse genetic protocols in which the GluN2B terminal valine (1,482) was deleted, leaving the last codon for Asp1471 before the stop codon (Supplementary Fig. 4a). In brief, 5.9 kb 5′ and 1.8 kb 3′ homology arms were used to construct the gene targeting vector for GluN2B terminal valine (1,482) deletion mutation. The targeting vector also contained a neomycin resistance cassette (pgk*-Neo*-pA) flanked by loxP sites at the 3′ of the mutation and diphtheria toxin A cassette for negative selection. A linearized targeting vector was electroporated into E14TG2a ES cells and positive clones were identified and validated by genomic southern blots with 5′ and 3′ flanking probes (data not shown). Positive ES clones were injected into C57/BL6 blastocysts to generate mice chimeric for the *Glun2b*^*2BdV*^ mutation. After germline transmission, the *Glun2b*^*2BdV*^ mutants were crossed with a Cre-deleter line and the neo cassette was removed. Inter-crossing heterozygous mice produced viable homozygous *Glun2b*^*2BdV/2BdV*^ knock-in mutants. The detailed phenotypic characterization of this mutant will be described elsewhere.

### Quantification of subunit molar ratios *in vivo*

Measurements of the molar ratios of GluN2A/GluN2B were performed using age-matched (P56-P69) total forebrain (cortex and hippocampus) samples. Quantifying the molar ratios of two different endogenous mouse proteins was achieved by using targeted mouse genetics to match an epitope for immuno-detection.

WT control with either *Glun2b*^*2A(CTR)/2A(CTR)*^ or *Glun2a*^*2B(CTR)/2B(CTR)*^ were used for two independent measurements of the GluN2A/GluN2B molar ratio (Fig. 4). In the first measurement of the GluN2A/GluN2B molar ratio was made using *Glun2b*^*2A(CTR)/2A(CTR)*^ mice, in which the CTD of GluN2B was replaced with that of GluN2A. Immunoblotting the amino-terminal domains showed this domain replacement caused no change in the total expression levels of GluN2A, GluN2B or GluN1 compared with WT. Next, using a GluN2A CTD specific monoclonal antibody gave a measurement of GluN2A in WT mice, and the sum of GluN2A and GluN2B subunits in *Glun2B*^*2A(CTR)/2A(CTR)*^ mice from which the molar ratio of GluN2A/GluN2B was calculated. *Glun2A*^*2B(CTR)/2B(CTR)*^ mice served as a control for the specificity of the GluN2A CTD antibody. The reciprocal measurement of the GluN2B CTD using *Glun2a*^*2B(CTR)/2B(CTR)*^ was also performed using a GluN2B CTD specific monoclonal antibody. Measurements were performed by densitometry of immunoblots using a dilution series (Fig. 4b) to ensure differences in band intensity corresponded to linear differences in the concentration of these epitopes.

### Primary neuronal culture

Primary neuronal cultures of cortical and hippocampal cells (Supplementary Fig. 1d) were prepared from E17.5 WT and *Glun1*^*TAP/TAP*^ embryos and cultured at 10^5^ cells cm^−2^ (for imaging) on poly-D-lysine coated glass cover-slips until they reached DIV7 (for 18–24 h 0.25 μM TSA treatment), DIV14 (for 8 h 10 μM 2-BP treatment). For biochemical analysis, cells were extracted in 100 μl 100:100:1 buffer H, buffer X (containing sodium deoxycholate at 1% w/v final concentration) and 10 kU benzonase at 5 °C for 20 min.

### Immunofluorescence confocal microscopy

Primary neurons were fixed with methanol, blocked with 3% bovine serum albumin (in 0.5% triton X-100, PBS) for 1 h at RT. Incubations with primary antibody in blocking buffer were for 1 h at RT, followed by 3 × 5 min wash in PBS. Incubations with secondary antibody in blocking buffer were for 2 h at RT, followed by 3 × 5 min wash in PBS and 3 × 5 s washes in de-ionized water. Cover slips were mounted on slides with antioxidants and DAPI (antifade, Invitrogen).

Digital photographs of 0.8–1 μm optical sections were collected at on a Zeiss 710 confocal microscope with × 63/1.4 numerical aperture objective.

### Immunohistochemistry

Homozygous Glun1^TAP/TAP^ and wild-type mice were anaesthetized with 60 mg g^−1^ avertin and perfused with 10 ml PBS, followed by 5 ml 4% paraformaldehyde at 2.5 ml min^−1^. Forebrains were dissected, post-fixed in 4% paraformaldehyde for 2–4 h and stored at 4 °C in 30% sucrose for up to one week. Sagittal sections (30 μm) were collected with a vibratome. Free-floating sections were incubated with 1:500 Rabbit Flag (Genscript, A000187) or 1:200 Rabbit GluN2A (Serotec, AHP1880) in buffer A (0.5% triton X-100, 100 mM NaCl, 50 mM tris.Cl pH 7.4 overnight at 4 °C. Following 3 × 10 min washes with buffer A at RT, sections were DAB stained with anti-rabbit-HRP using a kit (Vectastain) and mounted between cover slip and slide.

### Slice electrophysiology

Hippocampal slices (400-μm-thick) were prepared using standard techniques approved by the UCLA Institutional Animal Care and Use Committee. Slices were maintained in an interface-slice type chambers (at 30 °C) perfused with oxygenated (95% O_2_/5% CO_2_) artificial cerebrospinal fluid containing 124 mM NaCl, 4.4 mM KCl, 25 mM NaHCO_3_, 1 mM NaH_2_PO_4_, 2 mM CaCl_2_, 1.2 mM MgSO_4_ and 10 mM glucose. Techniques described elsewhere^48^ were used to record field excitatory postsynaptic potentials evoked by Schaffer collateral/commissural fibre stimulation in the hippocampal CA1 region. LTP was elicited using two trains of 100 Hz stimulation, each 1 s in duration, with an inter-train interval of 10 s. Whole-cell voltage-clamp techniques were used to record excitatory postsynaptic currents (EPSCs) evoked by Schaffer collateral fibre stimulation. Patch electrodes (4–6 MΩ) were filled with a solution containing 102 mM caesium gluconate, 17.5 mM CsCl, 10 mM TEA-Cl, 5 mM QX314, 4.0 mM Mg-ATP, 0.3 mM tris-GTP and 20 mM HEPES (pH=7.2), and slices were bathed in a modified artificial cerebrospinal fluid containing picrotoxin (100 μM), elevated concentrations of CaCl_2_ and MgSO_4_ (4 mM each) and 2.4 mM KCl. AMPAR-mediated and NMDAR-mediated components of evoked EPSCs were measured using current amplitudes 5 and 50 ms after EPSC onset, respectively. Double exponential fits to the decay of synaptic currents measured at +40 mV were used to calculate the weighted mean decay time constant for the decay of NMDAR-mediated currents^65^. All results are reported as mean±s.e.m. The results for the same experiment on multiple slices or cells from the same animal were averaged and the N used for statistical comparisons (two-tailed Student's *t*-tests) equals the number of animals in each group.

### Antibodies

Primary antibodies (dilutions for immunoblot in parenthesis: mouse Flag (Sigma, F3165) mouse GluN1 (1:1,000, Invitrogen, 32–0500), rabbit GluN1-1 (1:500, Millipore, AB9864), mouse GluN2A (1:1,000, BD Bioscience, 612287), rabbit GluN2A (1:500, Serotec, AHP1880), mouse GluN2B (1:1,000, BD Bioscience, 610416/7), rabbit GluN2B (1:500, Invitrogen, 71–8,600), mouse PSD95/3 (1:3,000, Thermo scientific, MA1–045), mouse PSD93 (1:2,500, Neuromab, 75-057), rabbit PSD95 (1:1,000. Abcam, ab18258), mouse GFP (Invitrogen, A11120), rabbit GFP (Invitrogen, A11122). Conjugated antibodies: mouse Flag-HRP (1:40,000, Sigma, A8592), goat mouse-HRP (1:20,000, Millipore, 12–349) and goat rabbit-HRP (1:20,000, Millipore, 12–348). Antibodies used for Fig. 7 are listed in Supplementary Data 1.

### SDS–PAGE immunoblot

Images in Figs 2b, 4a,b,d and 6h have been cropped for presentation. Full size images are presented in Supplementary Figs 6–8, respectively.

### Human brain samples

Three cortical samples were obtained from neurosurgical operations. Tissue was immediately chilled in ice for 30–40 min after which they were frozen at −70 °C. Tissue was not defrosted until used extraction and BNP. In all cases prior written informed consent had been obtained, and the study approved by the local regional ethics committee (Lothian Region Ethics Committee /2004/4/16).

### Animals

All animal experiments conformed to the British Home Office Regulations (Animal Scientific Procedures Act 1986; Project License PPL80/2,337 to Prof Seth Grant), local ethical approval, and NIH guidelines.

## Additional information

**Accession codes:** PRIDE database (http://www.ebi.ac.uk/pride/)^64^ with ProteomeXchange accession number: PXD000011.

**How to cite this article:** Frank, R. A. W. *et al*. NMDA receptors are selectively partitioned into complexes and supercomplexes during synapse maturation. *Nat. Commun.* 7:11264 doi: 10.1038/ncomms11264 (2016).

## Supplementary Material

Supplementary Information Supplementary Figures 1-8 and Supplementary Tables 1-2

Supplementary Data 1 Native supermolecular assembly screen
