# Supplementary material for: NMDA receptors are selectively partitioned into complexes and supercomplexes during synapse maturation
Source: Nat Commun. 2016 Apr 27;7:11264. doi: 10.1038/ncomms11264 (PMC5227094; doi:10.1038/ncomms11264)
Supplement: Supplementary Information — Supplementary Figures 1-8 and Supplementary Tables 1-2 [file ncomms11264-s1.pdf]

Supplementary Figure 1

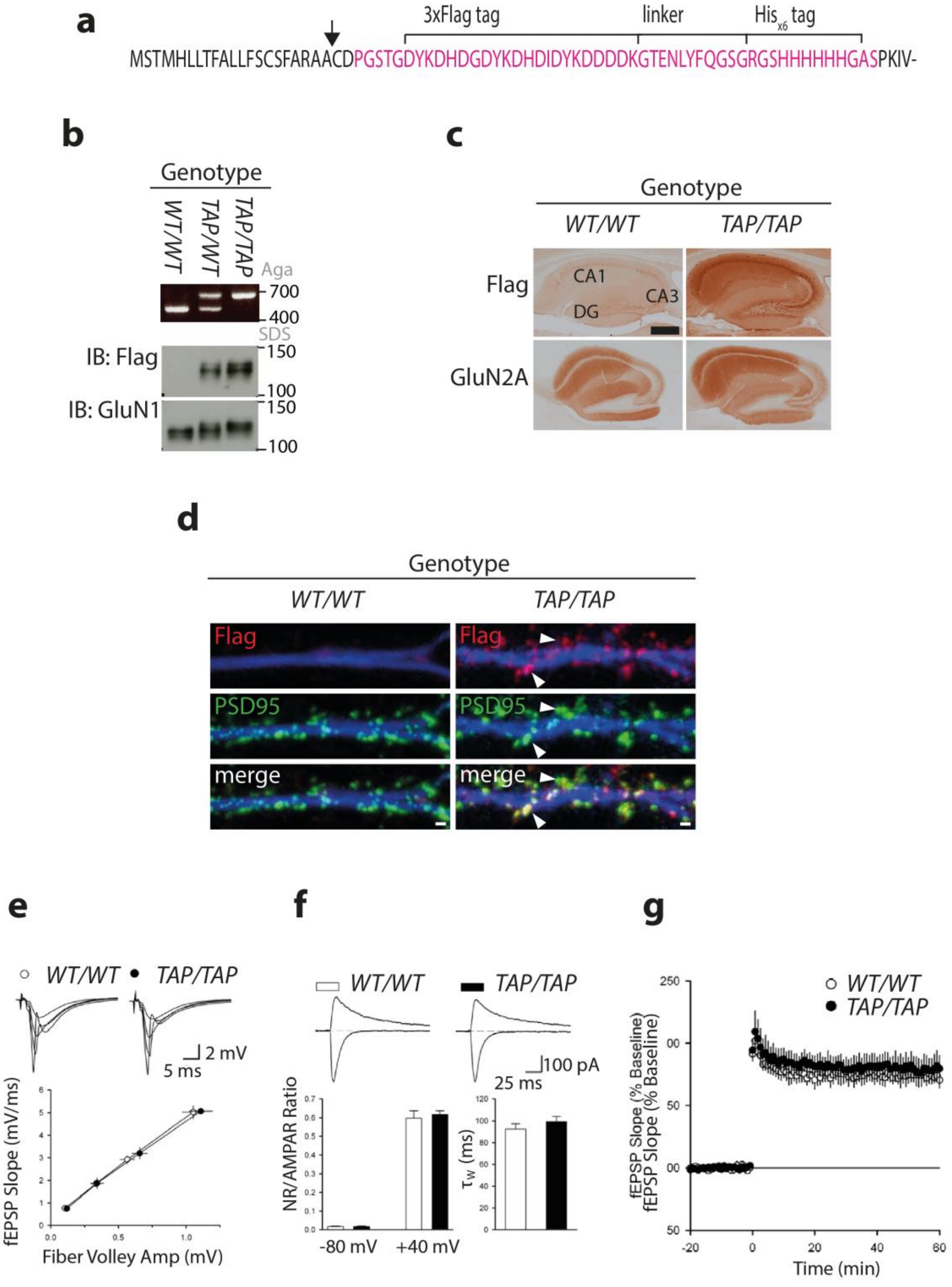

Supplementary Figure 1. Generation and validation of TAP-tagged GluN1 knockin mouse. To exclude the possibility of a mutant phenotype resulting from engineering TAP-GluN1, *Glun1*$^{TAP/TAP}$ mice were examined in biochemical, anatomical, and electrophysiological assays.

(a) The primary sequence of the engineered GluN1 N-terminus with the first 27 amino acids (in black) and inserted tandem affinity peptide (TAP)-tag (magenta). Arrow, predicted signal peptidase cleavage site; 3xFlag, 3 copies of Flag epitope; linker/TEV, protease cleavage site; His_{x6}, immobilized metal affinity (IMAC) tag.

(b) The detection of *Glun1*^{TAP} genotype by PCR and immunoblot. Upper panel, PCR genotyping products were separated on an agarose gel. Middle and lower panels, Flag (TAP-GluN1) and GluN1 immunoblot of total protein extract separated by SDS-PAGE.

(c) Sagittal sections of hippocampus from *Glun1*$^{TAP/TAP}$ and wild-type (WT) mice immunostained for Flag (top panels) and GluN2A (bottom panels). CA1, CA3, dentate gyrus (DG) subfields indicated. 0.5 mm scale bar. These data show the anatomical distribution of NMDAR subunits (GluN1 and GluN2A) was unchanged in *Glun1*$^{TAP/TAP}$ compared to WT.

(d) Fluorescence confocal microscopy photograph of a dendrite from a primary cultured neuron (DIV21) of WT (left panels) and *Glun1*$^{TAP/TAP}$ (right panels) immunostained with MAP2 (blue), Flag (red, top and bottom panels) and PSD95 (green, middle and bottom panels). White arrowheads indicate examples of co-localized PSD95 and Flag puncta. White line, 1 μm scale bar. These data show TAP-GluN1 were trafficked to synapses as expected.

(e) Input/output curves were generated by comparing presynaptic fibre volley amplitudes to the slope of the postsynaptic fEPSPs evoked by varying intensities of presynaptic fibre stimulation. Elicited fEPSPs were 25, 50, 75, or 100% of maximum fEPSP amplitude. Results are from 4 WT (12 slices) and 4 *Glun1*$^{TAP/TAP}$ mutant mice (12 slices). These data indicate basal AMPAR-mediated field EPSPs were normal in *Glun1*$^{TAP/TAP}$ mice.

(f) NMDA receptor/AMPA receptor ratios for evoked EPSCs elicited by Schaffer collateral fibre stimulation in CA1 pyramidal cells voltage-clamped at either -80 mV or + 40 mV. No significant difference was seen at either holding potential (-80 mV: $t(6) = 0.095$, $p = 0.93$); +40 mV: $t(6) = 0.446$, $p = 0.67$). The time constant of decay for currents recorded at +40 mV (weighted 2-exponential fit) was also not altered in *Glun1*$^{TAP/TAP}$ mice ($t(6) = 1.006$, $p = 0.35$). Results are from 11 cells from 4 WT mice and 10 cells from 4 *Glun1*$^{TAP/TAP}$ mice. These data indicate the NR-mediated component of evoked EPSCs was normal in *Glun1*$^{TAP/TAP}$ mice.

(g) Long-Term Potentiation (LTP) of synaptic transmission induced by 2 x 100 Hz/1sec (ITI = 10s). 60 min post-HFS fEPSPs were 173 ± 5% of baseline in WT slices (n = 6 slices from 3 mice) and 179 ± 10% of baseline in slices from *Glun1*$^{TAP/TAP}$ mice (n = 8 slices from 4 mice). $t(5) = 0.46$, $p = 0.66$. These data show normal NR-dependent LTP in *Glun1*$^{TAP/TAP}$ compared to WT.

Supplementary Figure 2

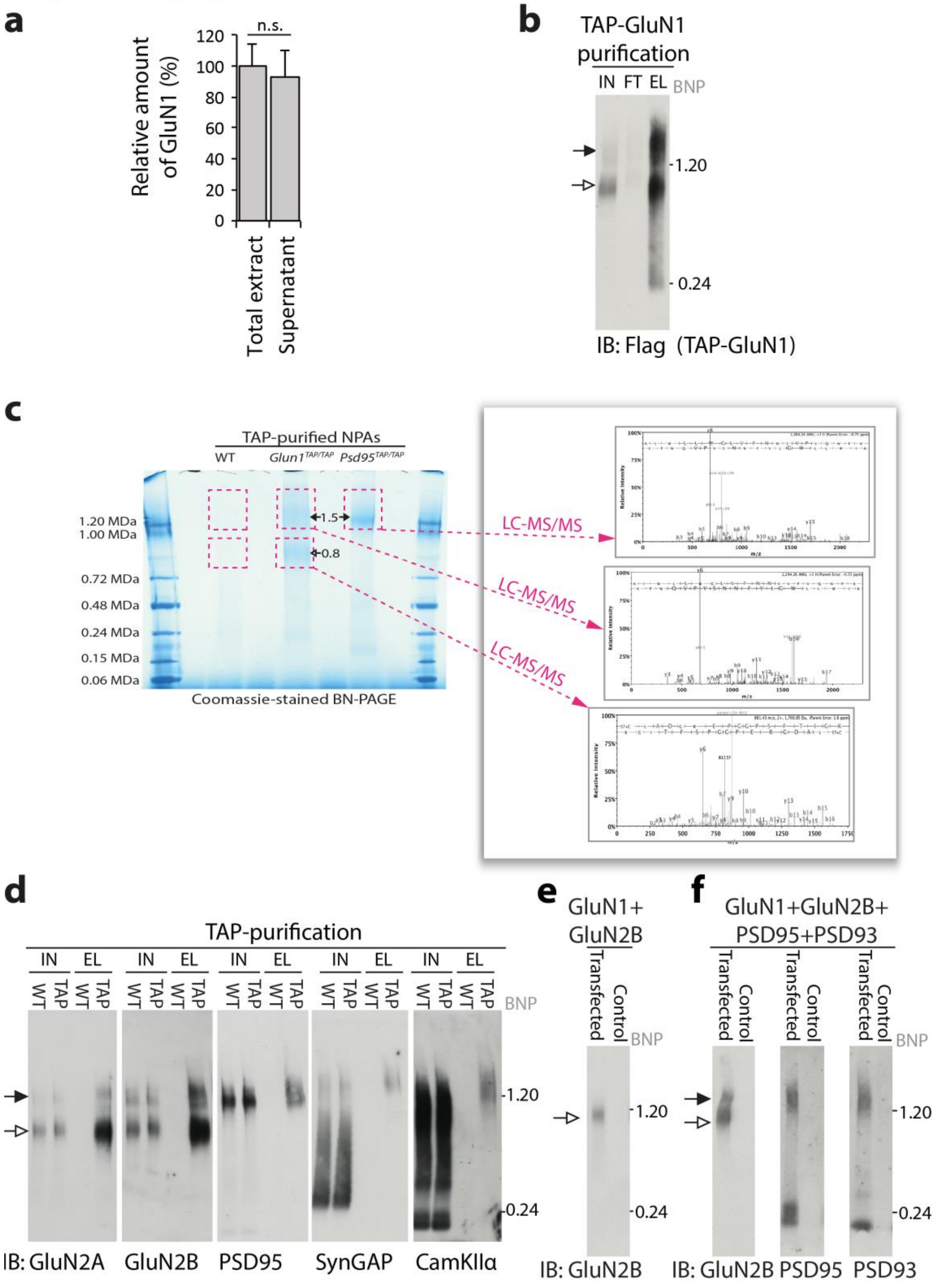

Supplementary Figure 2.

(a) Densitometric quantification of SDS immunoblots (see Fig. 2b, upper panel) shows after 120,000 x*g*. centrifugation for 40 minutes the amount of GluN1 in extract supernatant (n=3) is 93% of total extract (n=3). The difference between total extract and extract supernatant was not significant (P>0.05, mean and s.e.m shown). These data show the solubilization conditions for purification of TAP-tagged NMDARs extract nearly all NMDARs.

(b) BNP Flag (TAP-GluN1) immunoblot of TAP-purification from *Glun1*$^{TAP/TAP}$ mouse forebrain synaptoneurosomes. Lanes left to right: 'IN', extract supernatant; 'FT', flow-through; 'EL', eluate by peptide-antigen exchange elution) immunoblotted with Flag antibody. These data show the 1.5-NR and 0.8-NR remains intact during TAP-purification.

(c) Schematic of native proteomic approach (TAP-BNP-MS). Coomassie-stained BNP of Flag-affinity purifications yielding from left to right: negative control, TAP-GluN1 complexes, and PSD95-TAP complexes from wild-type (WT), *Glun1*$^{TAP/TAP}$ and *Psd95*$^{TAP/TAP}$ mice, respectively. Protein bands (corresponding to 0.8-NR, 1.5-NR and 1.5-PSD95 as indicated) were excised for trypsin digest, liquid chromatographic (LC) separation and mass spectrometric (MS/MS) analysis (see Supplementary Table 1 and 2). Equivalent gel pieces from wild-type lane were used as negative control samples for MS protein identifications. Triplicate experiments were used for analysis (see Methods).

(d) Validation of constituents identified in 0.8-NR and 1.5-NR by native proteomics (TAP-BNP-MS) by BNP immunoblot of GluN2A, GluN2B, PSD95, SynGAP, and CamKIIα. IN, forebrain extract supernatant; EL, eluate from Flag-affinity purification. Purification from wild-type (WT) and *Glun1*$^{TAP/TAP}$ (TAP) mice. These data show PSD95, SynGAP and CamKIIα were confined to 1.5-NR and were consistent with 0.8-NR corresponding to 'naked' NMDAR channel complexes. Thus, the detection of PSD95 and CamKIIα in 0.8-NR by BNP-MS (Supplementary Table 1) is likely to reflect the incomplete separation of 0.8-NR from 1.5-NR for the purposes of MS detection.

(e) Reconstitution of 0.8-NR in HEK293 cells transiently co-transfected with cDNAs encoding GluN1 and GluN2B, or control (empty expression vector only). BNP

immunoblot detected GluN2B in single complex of ~0.8 MDa in mass. These data indicate that 0.8-NR likely contains NMDAR subunits in addition to lipid and detergent in a surrounding micelle.
Open arrow indicates 0.8-NR.
Molecular weight in MDa shown on right.

(f) Reconstitution of the minimal core of 1.5-NR consisting of GluN1, GluN2B, PSD95 and PSD93. HEK293 cells were transiently co-transfected with cDNAs encoding GluN1, GluN2B, PSD95 and PSD93; or control (empty expression vector). BNP immunoblot detected from left to right: GluN2B, PSD95, and PSD93. These data show that when GluN1 and GluN2B are co-expressed with PSD95 and PSD93, they assemble into 0.8-NR and 1.5-NR. PSD95 and PSD93 expressed in HEK cells assemble into 1.5 MDa complexes and were also present in low molecular bands (>240 kDa), equivalent in mass to unassembled monomers.
Filled arrow indicates 1.5-NR and open arrow indicates 0.8-NR.
Molecular weight in MDa shown on right.

Supplementary Figure 3

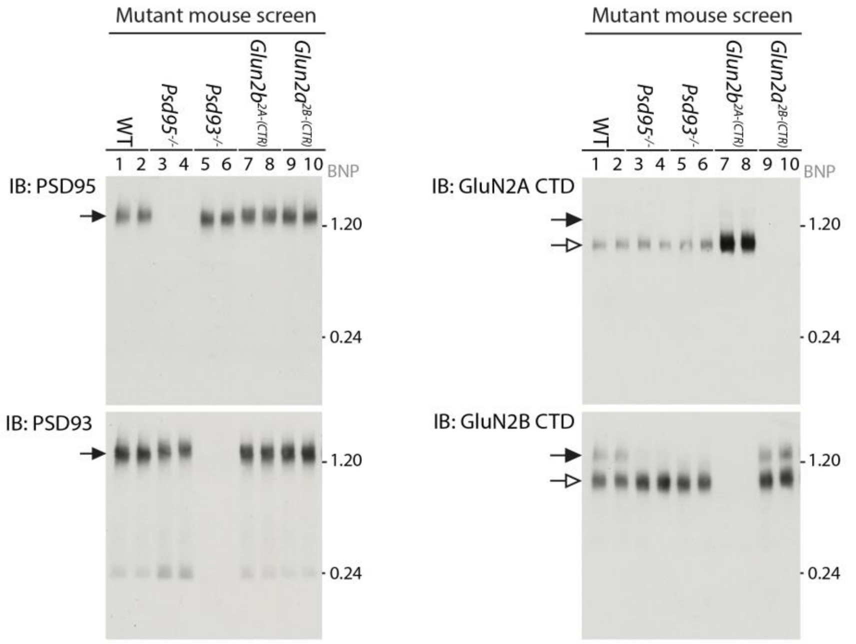

Supplementary Figure 3. BNP immunoblots of forebrain extracts from 4 mutant mouse strains. Each panel contains duplicates of WT (lane 1,2, duplicates), *Psd95*$^{-/-}$ (lane 3,4), *Psd93*$^{-/-}$ (lane 5,6), *Glun2b*$^{2A(CTR)/2A(CTR)}$ (lane 7,8), *Glun2a*$^{2B(CTR)/2B(CTR)}$ (lane 9,10).

Immunoblotting antibody is indicated below each panel (IB).

Filled arrow indicates ~1.5 MDa complexes and open arrow indicates 0.8-NR.

Molecular weight in MDa shown on right.

Representative results from triplicate experiments shown.

Supplementary Figure 4

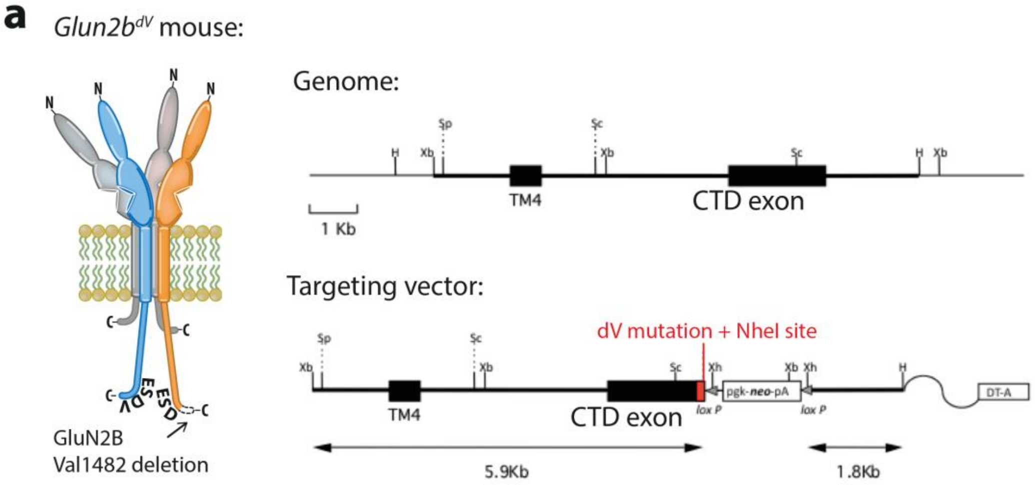

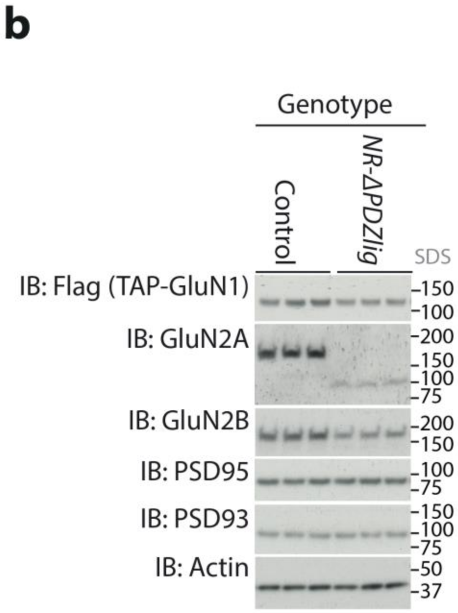

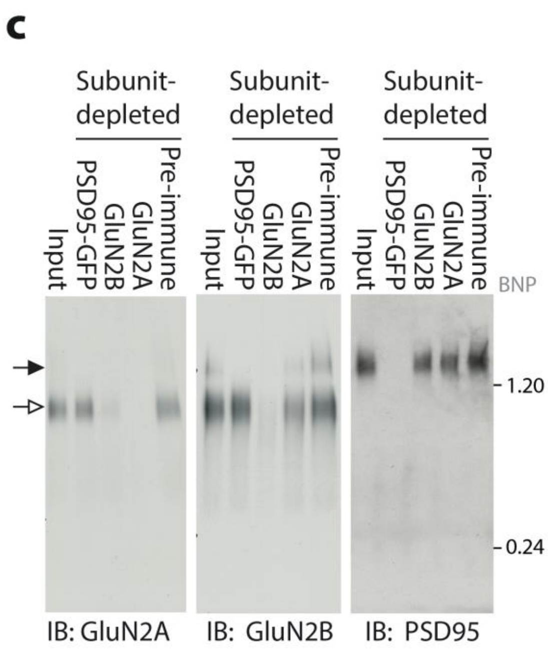

Supplementary Figure 4.

(a) *Left*, schematic of *Glun2b*^{dV} mutant mice. Arrow, indicates deletion of C-terminal Val1482 to disrupt ESDV motif/PDZ ligand in this mutant mouse. Cyan, GluN2A; Orange, GluN2B; grey, GluN1.

Right, schematic showing the construction of the targeting vector for generating the *Glun2b*^{dV} knockin mouse by homologous recombination in ES cells. See supplementary materials and methods for details. Schematic showing the construction of the targeting vector for generating the *Glun2b*^{dV} knockin mouse by homologous recombination in ES cells. See methods for details.

(b) Total expression of NMDAR subunits (GluN1, GluN2A, and GluN2B) was reduced in mice lacking GluN2 PDZ ligands. SDS-PAGE immunoblot of *NR- Δ PDZlig* (*Glun2b*$^{dV/dV}$/*Glun2a*$^{dC/dC}$/*Glun1*$^{TAP/TAP}$ triple knockin and controls (*Glun1*$^{TAP/TAP}$ only) forebrain extract.

Immunoblotting antibody is indicated at left of each panel (IB).

Molecular weight in KDa shown on right.

Representative results from triplicate experiments (separate animals) shown.

(c) Subunit depletion from *Glun1*$^{TAP/TAP}$/*Psd95*$^{GFP/GFP}$ double homozygous knockin forebrain extracts.

Left panel, BNP GluN2A immunoblot.

Middle panel, BNP GluN2B immunoblot.

Right panel, BNP PSD95 immunoblot.

Lanes left to right: Input, forebrain extract supernatant sample; PSD95-GFP depleted (with GFP antibody) sample; GluN2B, GluN2B-depleted sample; GluN2A, GluN2A-depleted sample; Pre-immune, negative control IgG. These data control for the completeness of GluN2, GluN2B, and PSD95-GFP removal from the sample by subunit-depletion in Fig. 3e.

Immunoblotting antibody is indicated (IB).

Filled arrow indicates 1.5-NR and open arrow indicates 0.8-NR.

Molecular weight in MDa shown on right.

Representative data from triplicate experiments shown.

Supplementary Figure 5

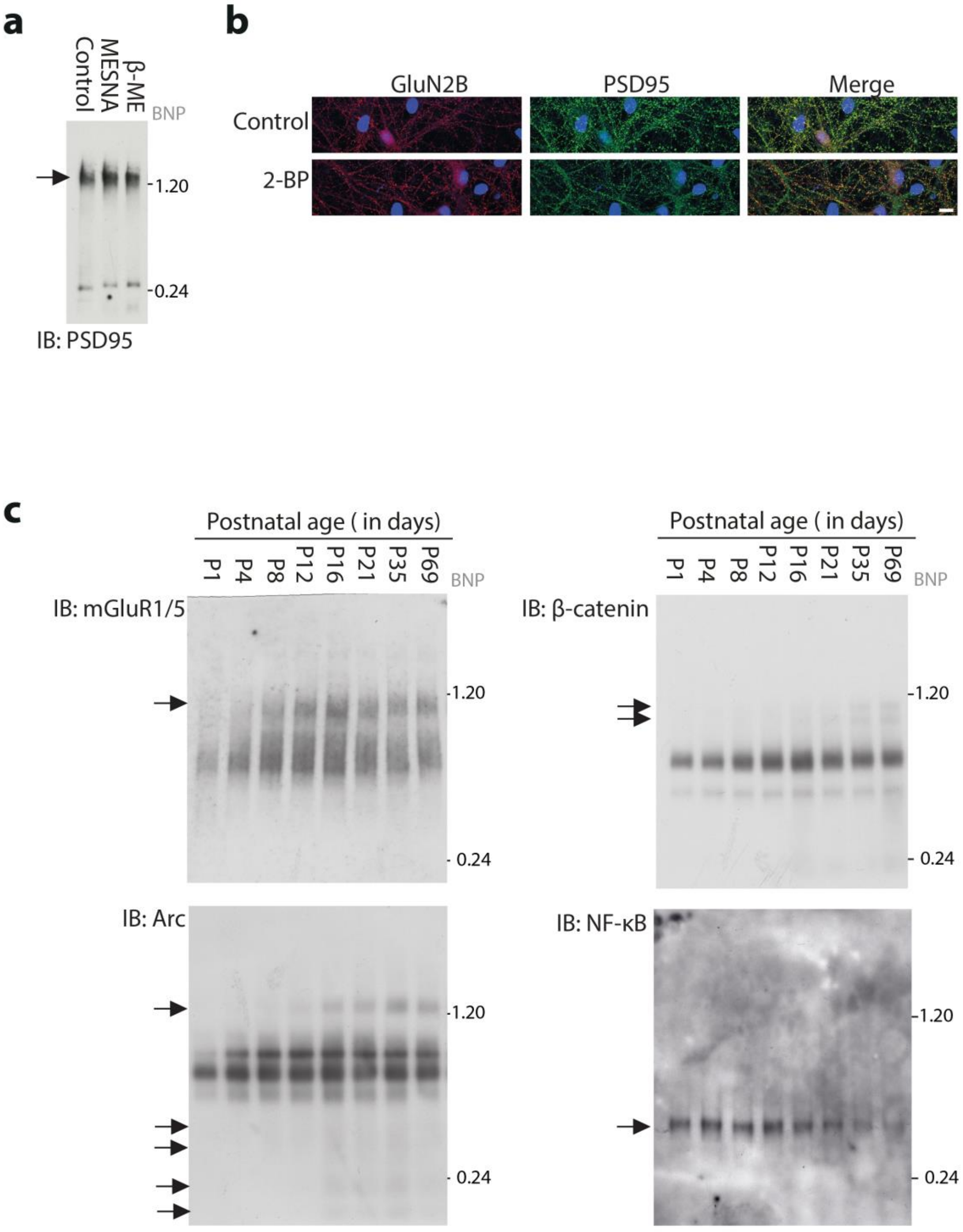

Supplementary Figure 5.

(a) In vitro depalmitoylation of forebrain extracts by thiol-exchange. From left to right: control (PBS), MESNA (40 mM 2-mercaptoethane sulphonate), β-ME (40 mM 2-mercaptoethanol) were added to forebrain extracts for 24 h before samples were analysed by BNP PSD95 immunoblot. These data are representative of three replicates. No change in the assembly of 1.5-PSD95 was observed. These data indicate palmitoylation is not required for maintaining PSD95 within 1.5 MDa supercomplexes.

(b) Inhibition of palmitoylation decreases the punctate distribution of PSD95.
Cultured primary cortical neurons (DIV14) were treated with DMSO (top panels) 10 µM 2-BP (bottom panels) for 8 h before cells were fixed and analysed by immunofluorescence microscopy to detect PSD95 (green), GluN2B (red). counter stained. Nuclei were stained with DAPI (blue).

(c) Distinct temporal profiles of assembly during postnatal development.
Postnatal development of mGluR1/5, β–catenin, Arc, and NF-κB complexes detected by BNP immunoblot of total forebrain extract samples taken at postnatal days: P1, P4, P8, P12, P16, P21, P35 and P69. Arrows indicate complexes whose assembly was regulated during postnatal development.

Supplementary Figure 6

Related to Fig. 2b.

Top panel.

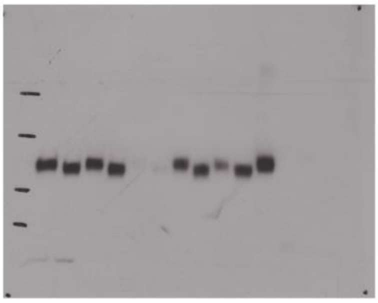

Second from top panel.

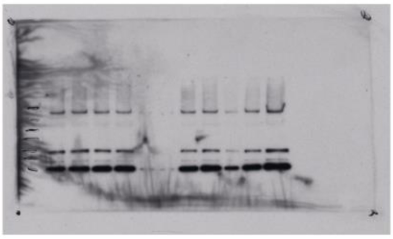

Third from top panel.

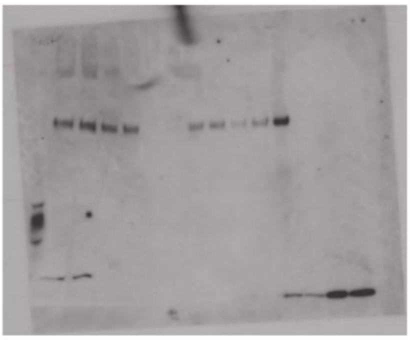

Third from top panel.

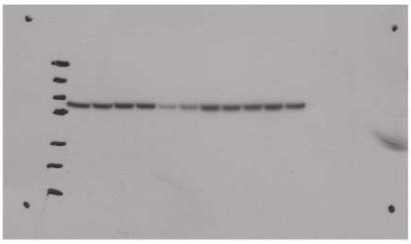

Bottom panel.

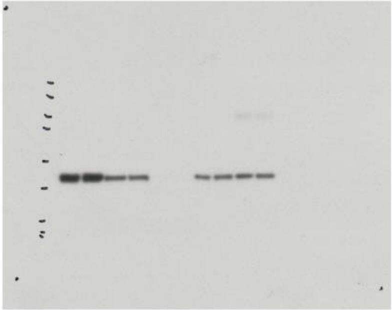

Supplementary Figure 6.

Full size SDS-PAGE immunoblots related to Fig. 2b. MW markers (Biorad) when present are shown on left of immunoblot from top to bottom: 200 kDa, 150 kDa, 100 kDa, 75 kDa, 50 kDa, 37 kDa, 25 KDa, 20 kDa, 15 kDa, 10 kDa.

Supplementary Figure 7

Related to Fig. 4a.

Top panel.

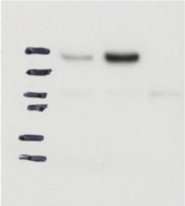

Second from top panel.

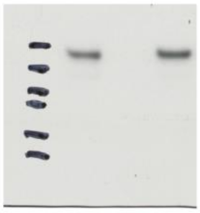

Third from top panel.

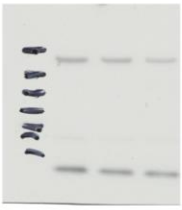

Third from top panel.

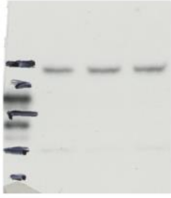

Bottom panel.

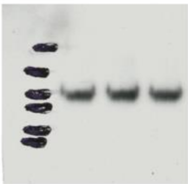

Related to Fig. 4b.

Top panel.

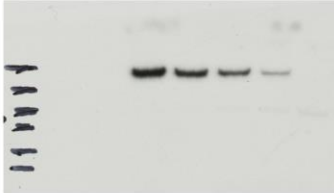

Bottom panel.

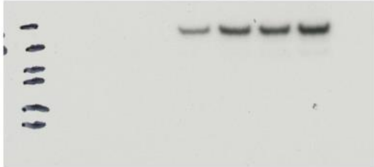

Related to Fig. 4d.

Top-left panel.

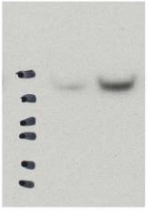

Bottom-left panel.

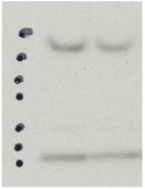

Top-middle panel.

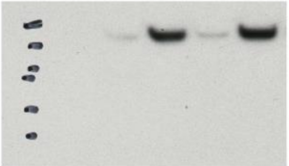

Bottom-middle panel.

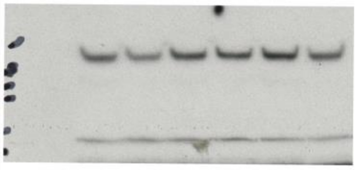

Top-right panel.

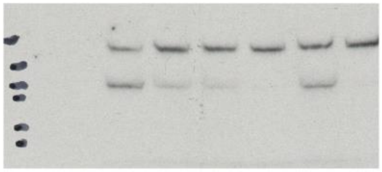

Bottom-right panel.

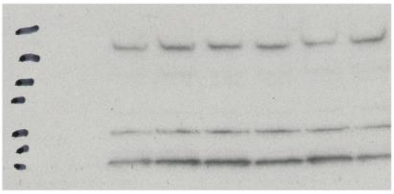

Supplementary Figure 7.

Full size SDS-immunoblots related to Fig. 4a, b, and d. MW markers (Biorad) when present are shown on left of immunoblot from top to bottom: 200 kDa, 150 kDa, 100 kDa, 75 kDa, 50 kDa, 37 kDa, 25 KDa, 20 kDa, 15 kDa, 10 kDa.

Supplementary Figure 8

Related to Fig. 6g.

Top panel.

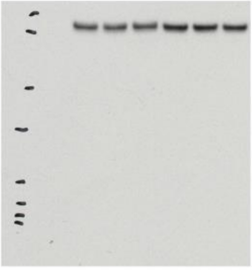

Middle panel.

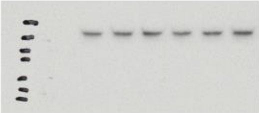

Bottom panel.

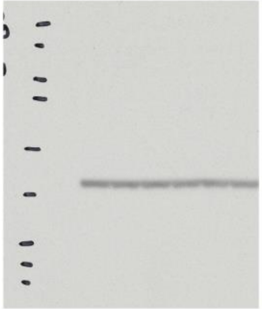

Related to Supplementary Fig. 1b.

Top panel.

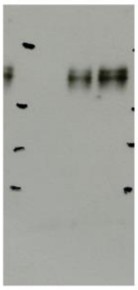

Bottom panel.

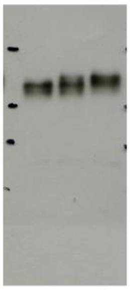

Related to Supplementary Fig. 4b.

Top panel.

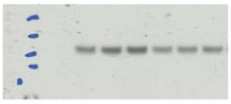

Second from top panel.

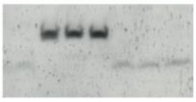

Fourth from top panel.

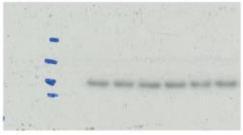

Third from top panel.

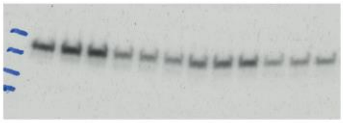

Third from top panel.

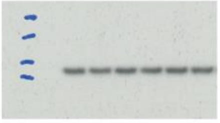

Bottom panel.

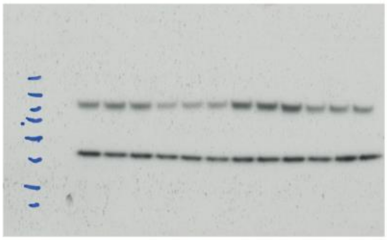

Supplementary Figure 8.

Full size SDS-immunoblots related to Fig. 6g, Supplementary Fig. 1b and 4b. MW markers (Biorad) when present are shown on left of immunoblot from top to bottom: 200 kDa, 150 kDa, 100 kDa, 75 kDa, 50 kDa, 37 kDa, 25 KDa, 20 kDa, 15 kDa, 10 kDa.

Supplementary Table 1. Proteins identified in native NMDARs by TAP-BNP-MS. Samples were purified in triplicate from WT (control) and TAP-GluN1 knock-in mice and separated by BNP (see Fig. 2c and Supplementary Fig. 2c). Coommassie-stained BNP gel bands were excised, trypsin-digested and analysed by LC-MS/MS. Proteins classified by function. Detection threshold set to minimize apparent cross-contamination: ≥4 unique peptides and ≥2 replicate samples of ≥2 unique peptides. * indicates proteins not shared between 1.5-NR and 0.8-NR. All raw data deposited at the PRIDE database, accession numbers: 27077-27080

	**1.5-NR**		**0.8-NR**	
	Name	Uniprot ID	Name	Uniprot ID
Neurotransmitter receptors:				
	GluN2B	NMDE2_MOUSE	GluN2B	NMDE2_MOUSE
	GluN1	A2AI21_MOUSE	GluN1	A2AI21_MOUSE
	GluN2A	NMDE1_MOUSE	GluN2A	NMDE1_MOUSE
	GluN3A	A2AIR5_MOUSE	GluN3A	A2AIR5_MOUSE
	GluN2D	C9K0Z5_MOUSE	GluN2D	C9K0Z5_MOUSE
*	GluA2	C9K0Z0_MOUSE	-	-
Adaptor / scaffold proteins				
	PSD95	DLG4_MOUSE	PSD95	DLG4_MOUSE
	PSD93	DLG2_MOUSE	PSD93	DLG2_MOUSE
	Sap102	Q80TH1_MOUSE	Sap102	Q80TH1_MOUSE
*	Dlgap1	DLGP1_MOUSE	-	-
*	IQSec2	IQEC2_MOUSE	-	-
*	Sap97	DLG1_MOUSE	-	-
*	Dlgap4	B7ZNS2_MOUSE	-	-
*	Dlgp1	DLGP2_MOUSE	-	-
*	IQSec1	IQEC1_MOUSE	-	-
*	Dlgap3	B1AS06_MOUSE	-	-
*	Anks1	ANS1B_MOUSE	-	-
*	Densin-180	LRRC7_MOUSE	-	-
Second messenger signaling:				
	CamKIIα	KCC2A_MOUSE	CamKIIα	KCC2A_MOUSE
*	SynGAP	SYGP1_MOUSE	-	-
*	CamKIIβ	KCC2B_MOUSE	-	-
*	Dock3	DOCK3_MOUSE	-	-
Adhesion proteins:				
*	ADAM22	ADA22_MOUSE	-	-
*	Bai3	BAI3_MOUSE	-	-
Ion channels:				
	Vdac1	VDAC1_MOUSE	Vdac1	VDAC1_MOUSE
*	Kcnj4	IRK4_MOUSE	-	-
*	Vdac3	VDAC3_MOUSE	-	-
Transporters:				
	Slc25a4	ADT1_MOUSE	Slc25a4	ADT1_MOUSE

	ATP1a3	AT1A3_MOUSE		ATP1a3	AT1A3_MOUSE
*	PMCA2	AT2B2_MOUSE	*	Slc25a3	MPCP_MOUSE
	-	-	*	Slc25a5	ADT2_MOUSE
Cytoskeletal proteins:					
	Tubulin-α4	TBA4A_MOUSE		Tubulin-α4	TBA4A_MOUSE
	Tubulin-β4	TBB4_MOUSE		Tubulin-β4	TBB4_MOUSE
*	Myosin-Va	MYO5A_MOUSE	*	Actin-γ	ACTG_MOUSE
			*	Cap2	CAP2_MOUSE
Other:					
	Hsp71	HSP7C_MOUSE		Hsp71	HSP7C_MOUSE
	Hsp70	A2AUF6_MOUSE		Hsp70	A2AUF6_MOUSE
	ATP5a1	ATPA_MOUSE		ATP5a1	ATPA_MOUSE
	Plp	MYPR_MOUSE		Plp	MYPR_MOUSE
*	ATP5c1	ATPG_MOUSE		-	-
*	Erlin2	ERLN2_MOUSE		-	-

Supplementary Table 2. Proteins identified in ~1.5 MDa complexes by TAP-BNP-MS. Samples were purified in triplicate from WT (control), TAP-GluN1 and PSD95-TAP knock-in mice and separated by BNP (see Fig. 2c and Supplementary Fig 2c). Coomassie-stained BNP gel bands were excised, trypsin-digested and analysed by LC-MS/MS. Proteins classified by function. Detection threshold: ≥2 unique peptides and ≥1 unique peptide in ≥2 replicate samples. * indicates proteins not shared between TAP-GluN1 and PSD95-TAP complexes. All raw data deposited at the PRIDE database, accession numbers: 27077-27080

**WT (control)**		**TAP-GluN1**		**PSD95-TAP**	
Name	Uniprot ID	Name	Uniprot ID	Name	Uniprot ID
Neurotransmitter receptors:					
-	-	GluN2B	NMDE2_MOUSE	GluN2B	NMDE2_MOUSE
-	-	GluN1	A2AI21_MOUSE	GluN1	A2AI21_MOUSE
-	-	GluN2A	NMDE1_MOUSE	GluN2A	NMDE1_MOUSE
-	-	GluA2	C9K0Z0_MOUSE	GluA2	C9K0Z0_MOUSE
-	-	GluN2D	C9K0Z5_MOUSE	GluN2D	C9K0Z5_MOUSE
-	-	* GluN3A	A2AIR5_MOUSE	* GluA1	GRIA1_MOUSE
-	-	-	-	* GluA3	A2VDF5_MOUSE
-	-	-	-	* GluK2	GRIK2_MOUSE
-	-	-	-	* GluK5	GRIK5_MOUSE
-	-	-	-	* GluA4	GRIA4_MOUSE
Adaptors / scaffold proteins:					
-	-	PSD93	DLG2_MOUSE	PSD93	DLG2_MOUSE
-	-	PSD95	DLG4_MOUSE	PSD95	DLG4_MOUSE
-	-	IQSec2	IQEC2_MOUSE	IQSec2	IQSEC_MOUSE
-	-	Dlgap1	DLGP1_MOUSE	Dlgap1	DLGP1_MOUSE
-	-	Sap97	DLG1_MOUSE	Sap97	DLG1_MOUSE
-	-	Dlgap4	B7ZNS2_MOUSE	Dlgap4	B7ZNS2_MOUSE
-	-	Dlgp1	DLGP2_MOUSE	Dlgp1	DLGP2_MOUSE
-	-	Dlgap3	B1AS06_MOUSE	Dlgap3	B1AS06_MOUSE
-	-	IQSec1	IQEC1_MOUSE	IQSec1	IQEC1_MOUSE
-	-	Ablim1	ABLM1_MOUSE	Ablim1	ABLM1_MOUSE
-	-	Anks1	ANS1B_MOUSE	Anks1	ANS1B_MOUSE
-	-	Sap102	Q80TH1_MOUSE	Sap102	Q80TH1_MOUSE
-	-	Begain	BEGIN_MOUSE	Begain	BEGIN_MOUSE
-	-	Densin-180	LRRC7_MOUSE	Densin-180	LRRC7_MOUSE
-	-	-	-	* Baiap2	BAIP2_MOUSE
Second messenger signalling:					
-	-	SynGAP	SYGP1_MOUSE	SynGAP	SYGP1_MOUSE
-	-	CamKIIα	KCC2A_MOUSE	CamKIIα	KCC2A_MOUSE
-	-	Cypin	GUAD_MOUSE	Cypin	GUAD_MOUSE
-	-	AP2-μ	AP2M1_MOUSE	AP2-μ	AP2M1_MOUSE
-	-	CamKIIβ	KCC2B_MOUSE	CamKIIβ	KCC2B_MOUSE
-	-	Ubiquitin	UBB_MOUSE	Ubiquitin	UBB_MOUSE
-	-	Gnao1	GNAO_MOUSE	Gnao1	GNAO_MOUSE
-	-	* Dock3	DOCK3_MOUSE	* Arc	ARC_MOUSE
-	-	-	-	* Rab3a	RAB3A_MOUSE
-	-	-	-	* Kalirin	KALRN_MOUSE
-	-	-	-	* Citron	CTRO_MOUSE
-	-	-	-	* DnaJc5	DNJC5_MOUSE
-	-	-	-	* RGS17	RGS17_MOUSE
-	-	-	-	* Dusp-10	DUS10_MOUSE
Adhesion proteins:					
-	-	ADAM22	ADA22_MOUSE	ADAM22	ADA22_MOUSE
-	-	Lgi1	LGI1_MOUSE	Lgi1	LGI1_MOUSE
-	-	Bai3	BAI3_MOUSE	Bai3	BAI3_MOUSE
-	-	Gpr123	Q52KJ6_MOUSE	Gpr123	Q8C4E9_MOUSE
-	-	Prr7	PRR7_MOUSE	Prr7	PRR7_MOUSE
-	-	-	-	* Neurexin1	NRX1A_MOUSE
-	-	-	-	* Il1rapl1	B1ASU0_MOUSE
-	-	-	-	* Ildr2	B5TVM2_MOUSE
-	-	-	-	* Bai2	BAI2_MOUSE
Ion channels:					
-	-	Kcnj4	IRK4_MOUSE	Kcnj4	IRK4_MOUSE
-	-	Vdac1	VDAC1_MOUSE	Vdac1	VDAC1_MOUSE
-	-	Vdac2	VDAC2_MOUSE	Vdac2	VDAC2_MOUSE
-	-	Vdac3	VDAC3_MOUSE	Vdac3	VDAC3_MOUSE

-	-		-	-	*	Kcna2	B2RS05_MOUSE
-	-		-	-	*	Ncnab2	KCAB2_MOUSE
-	-		-	-	*	Cacng2	CCG2_MOUSE
-	-		-	-	*	Kcna4	KCNA4_MOUSE
-	-		-	-	*	Kcnj2	IRK2_MOUSE
-	-		-	-	*	Kcnj10	IRK10_MOUSE
-	-		-	-	*	Cacng3	CCG3_MOUSE
-	-		-	-	*	Kcnj16	IRK16_MOUSE
Transporters:							
-	-		Slc25a4	ADT1_MOUSE		Slc25a4	Slc25a4
-	-		Slc25a3	MPCP_MOUSE		Slc25a3	Slc25a3
-	-		Slc25a5	ADT2_MOUSE		Slc25a5	Slc25a5
-	-		Slc25a11	M2OM_MOUSE		Slc25a11	Slc25a11
-	-	*	ATP1a3	AT1A3_MOUSE	*	Eaa2	Eaa2
-	-	*	PMCA2	AT2B2_MOUSE		-	-
Cytoskeletal proteins:							
-	-		Tubulin-α4	TBA4A_MOUSE		Tubulin-α4	TBA4A_MOUSE
-	-		Tubulin-β4	TBB4_MOUSE		Tubulin-β4	TBB4_MOUSE
-	-		Actin-γ	ACTG_MOUSE		Actin-γ	ACTG_MOUSE
-	-	*	Myosin-Va	MYO5A_MOUSE	*	Enah	ENAH_MOUSE
Protein of unknown function:							
-	-		-	-	*	FAM81A	FA81A_MOUSE
-	-		-	-	*	Fbxl17	Q7TNS5_MOUSE
-	-		-	-	*	FAM163B	F163B_MOUSE
Other:							
IgG	IGH1M_MOUSE		Hsp71	HSP7C_MOUSE		Hsp71	HSP7C_MOUSE
-	-		ATP5a1	ATPA_MOUSE		ATP5a1	ATPA_MOUSE
-	-		IgG	IGH1M_MOUSE		IgG	IGH1M_MOUSE
-	-		Plp	MYPR_MOUSE		Plp	MYPR_MOUSE
-	-		ATP5c1	ATPG_MOUSE		ATP5c1	ATPG_MOUSE
-	-		Hsp70	A2AUF6_MOUSE		Hsp70	A2AUF6_MOUSE
-	-	*	Rpl6	RL6_MOUSE	*	Prdx5	PRDX5_MOUSE
-	-	*	Erlin2	ERLN2_MOUSE	*	ATPp5o	ATPO_MOUSE
-	-	*	Hexokinase	HXK1_MOUSE	*	Glul	GLNA_MOUSE
-	-		-	-	*	ATP5l	ATP5L_MOUSE
-	-		-	-	*	LSm11	LSM11_MOUSE
-	-		-	-	*	Immt	IMMT_MOUSE
-	-		-	-	*	Igκc	A2NHM3_MOUSE
-	-		-	-	*	Ndufa4	NDUA4_MOUSE
-	-		-	-	*	Brp44	BR44_MOUSE
-	-		-	-	*	Mgst3	MGST3_MOUSE
-	-		-	-	*	Gpx4	GPX41_MOUSE
-	-		-	-	*	Sucla2	SUCB1_MOUSE
